# Probabilistic modeling of personalized drug combinations from integrated chemical screen and molecular data in sarcoma

**DOI:** 10.1186/s12885-019-5681-6

**Published:** 2019-06-17

**Authors:** Noah E. Berlow, Rishi Rikhi, Mathew Geltzeiler, Jinu Abraham, Matthew N. Svalina, Lara E. Davis, Erin Wise, Maria Mancini, Jonathan Noujaim, Atiya Mansoor, Michael J. Quist, Kevin L. Matlock, Martin W. Goros, Brian S. Hernandez, Yee C. Doung, Khin Thway, Tomohide Tsukahara, Jun Nishio, Elaine T. Huang, Susan Airhart, Carol J. Bult, Regina Gandour-Edwards, Robert G. Maki, Robin L. Jones, Joel E. Michalek, Milan Milovancev, Souparno Ghosh, Ranadip Pal, Charles Keller

**Affiliations:** 1 Children’s Cancer Therapy Development Institute, 12655 SW Beaverdam Road-West, Beaverton, OR 97005 USA; 2 Electrical and Computer Engineering, Texas Tech University, Lubbock, TX 79409 USA; 3 Department of Pediatrics, Oregon Health & Science University, Portland, OR 97239 USA; 4 Knight Cancer Institute, Oregon Health & Science University, Portland, OR 97239 USA; 5 Champions Oncology, Baltimore, MD 21205 USA; 6 Royal Marsden Hospital and Institute of Cancer Research, London, SW3 6JJ UK; 7 Department of Pathology, Oregon Health & Science University, Portland, OR 97239 USA; 8 Center for Spatial Systems Biomedicine Department of Molecular and Medical Genetics, Oregon Health & Science University, Portland, OR 97239 USA; 9 Department of Epidemiology and Biostatistics, University of Texas Health Science Center San Antonio, San Antonio, TX 78229 USA; 10 Department of Orthopedic Surgery, Oregon Health & Science University, Portland, OR 97239 USA; 11 Department of Pathology, Sapporo Medical University School of Medicine, Sapporo, 060-8556 Japan; 12 Department of Orthopaedic Surgery, Faculty of Medicine, Fukuoka University, Fukuoka, 814-0180 Japan; 13 The Jackson Laboratory, Bar Harbor, ME 04609 USA; 14 Department of Pathology & Laboratory Medicine, UC Davis Health System, Sacramento, CA 95817 USA; 15 Sarcoma Program, Zucker School of Medicine at Hofstra/Northwell & Cold Spring Harbor Laboratory, Long Island, NY 10142 USA; 16 Carlson College of Veterinary Medicine, Oregon State University, Corvallis, OR 97331 USA; 17 Department of Mathematics and Statistics, Texas Tech University, Lubbock, TX 79409 USA; 18 Department of Otolaryngology – Head and Neck Surgery, Oregon Health & Science University, Portland, OR 97239 USA; 19 Hôpital Maisonneuve-Rosemont, Montreal, H1T 2M4 Canada; 20 Omics Data Automation, 12655 SW Beaverdam Road, Beaverton, OR 97005 USA

**Keywords:** Personalized therapy, Combination therapy, Artificial intelligence and machine learning, Pediatric cancer, Sarcoma, Drug screening, High-throughput sequencing, Computational modeling

## Abstract

**Background:**

Cancer patients with advanced disease routinely exhaust available clinical regimens and lack actionable genomic medicine results, leaving a large patient population without effective treatments options when their disease inevitably progresses. To address the unmet clinical need for evidence-based therapy assignment when standard clinical approaches have failed, we have developed a probabilistic computational modeling approach which integrates molecular sequencing data with functional assay data to develop patient-specific combination cancer treatments.

**Methods:**

Tissue taken from a murine model of alveolar rhabdomyosarcoma was used to perform single agent drug screening and DNA/RNA sequencing experiments; results integrated via our computational modeling approach identified a synergistic personalized two-drug combination. Cells derived from the primary murine tumor were allografted into mouse models and used to validate the personalized two-drug combination.

Computational modeling of single agent drug screening and RNA sequencing of multiple heterogenous sites from a single patient’s epithelioid sarcoma identified a personalized two-drug combination effective across all tumor regions. The heterogeneity-consensus combination was validated in a xenograft model derived from the patient’s primary tumor.

Cell cultures derived from human and canine undifferentiated pleomorphic sarcoma were assayed by drug screen; computational modeling identified a resistance-abrogating two-drug combination common to both cell cultures. This combination was validated in vitro via a cell regrowth assay.

**Results:**

Our computational modeling approach addresses three major challenges in personalized cancer therapy: synergistic drug combination predictions (validated in vitro and in vivo in a genetically engineered murine cancer model), identification of unifying therapeutic targets to overcome intra-tumor heterogeneity (validated in vivo in a human cancer xenograft), and mitigation of cancer cell resistance and rewiring mechanisms (validated in vitro in a human and canine cancer model).

**Conclusions:**

These proof-of-concept studies support the use of an integrative functional approach to personalized combination therapy prediction for the population of high-risk cancer patients lacking viable clinical options and without actionable DNA sequencing-based therapy.

**Electronic supplementary material:**

The online version of this article (10.1186/s12885-019-5681-6) contains supplementary material, which is available to authorized users.

## Background

Despite decades of advancements in cancer treatment, over 600,000 patients with solid tumors die annually in North America [1], including approximately 5000 sarcoma-related deaths. The population of high-risk, late-stage, recurrent, rare or refractory cancer patients who have exhausted standard clinical pathways and lack further treatment options represents a major unmet clinical need. Currently, DNA sequencing of tumors for druggable mutations leaves approximately 60% of patients without an actionable result [2, 3]. Additionally, in many cases, single drug therapy fails to provide sustainable disease control [4]. A critical missing element in personalized cancer therapy design is the lack of effective methodologies for model-based prediction, design, and prioritization of patient-specific drug *combinations*, especially in the presence of limited tumor tissue material.

Numerous approaches to computational modeling of drug sensitivity and therapy assignment exist, in part to address ambiguity in DNA sequencing results [2, 5]. These approaches are primarily based on gene expression [6], or a combination of genomic and epigenomic data [7]. For instance, 1) integrative genomic models using Elastic Net regression techniques have been developed from large datasets such as the Cancer Cell Line Encyclopedia (CCLE) [8] database; 2) integrative models using Random Forests with Stacking [9, 10] to integrate multiple genetic data sets for sensitivity prediction; and 3) a team science based sensitivity prediction challenge produced independent models integrating multiple data types for sensitivity prediction [11]; despite 44 individual models and a “wisdom of crowds” approach merging the top-ranked predictive models together, none of the approaches surpassed 70% predictive accuracy [11] falling short of a reasonable accuracy threshold for clinical utility. Some recent work has focused on the use of functional data for therapy selection, such as 1) the use of microfluidics to test multiple drugs efficiently on primary patient samples [12], 2) the use of shRNA libraries to predict drug combinations for heterogenous tumor populations [13], and 3) a re-analysis of the CCLE database used machine learning models integrating functional response data to improve sensitivity prediction accuracy over molecular data-based Elastic Net models [14]. Integration of functional data may improve overall predictive accuracy over solely molecular data-based predictive models, especially for individual patient samples, emphasizing the need for improved drug sensitivity prediction to enable patient-specific therapy design.

**Fig. 1:**
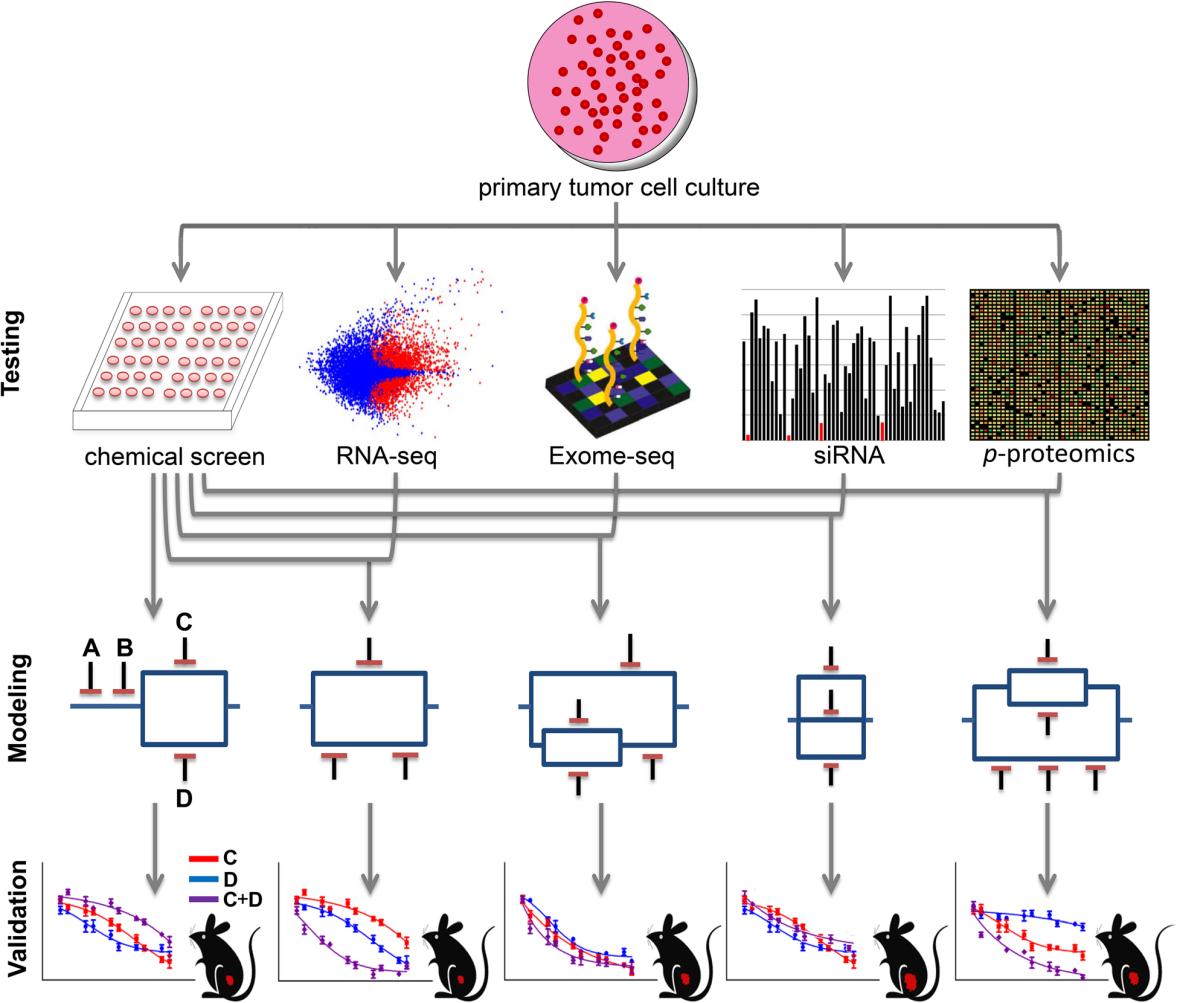
Schematic representation of experimental and computational approach to personalized combination targeted therapy predictions. Following tumor extraction and culture establishment, biological data is generated (e.g., chemical screening, transcriptome sequencing, exome sequencing, siRNA interference screening and phosphoproteomic analysis) and used as input for PTIM modeling. To briefly explain the graphical model representation, targets A and B denote two independent single points of failure. Targets C and D denote parallel targets, which independently are not predicted to be effective, but together will be synergistic and lead to significant cell growth inhibition. Targets A, B, and the C-D parallel block are in series and may target independent pathways. Series blocks, when inhibited together, may abrogate cancer resistance mechanisms by knockdown of independent pathways. Model sensitivity scores for gene target combinations are used to design and rank follow-up in vitro validation and in vivo validation experiments. The “Exome-Seq” representative images was adapted from an image on the Wikipedia Exome sequencing article originally created by user SarahKusala and available under Creative Commons 3.0 license. An unaltered portion of the image was used. The mouse image used is public domain and accessed through Bing image search at the following weblink: http://img.res.meizu.com/img/download/uc/27/83/20/60/00/2783206/w100h100

To address the need for accurate prediction of drug sensitivity and design of multi-drug combinations, we previously developed a functional drug sensitivity-based modeling approach termed Probabilistic Target Inhibition Maps (PTIMs) [14–17]. The base PTIM methodology integrates quantified drug-target inhibition information (EC_50_ values) and log-scaled experimental drug sensitivities (IC_50_ values) to identify mechanistic target combinations explaining drug sensitivity data. PTIM modeling improved predictive accuracy over Elastic Net models from the CCLE dataset [14], and has guided in silico validation experiments from primary canine osteosarcoma cell models [14, 16–18] and in vitro validation experiments [19] on diffuse intrinsic pontine glioma (DIPG) cell models. Herein, we present proof-of-concept validation experiments of the integrative PTIM pipeline (Fig. 1) using soft tissue sarcoma as a paradigm. Each validation experiment applies PTIM combination therapy design to address one of three critical unmet needs in cancer treatment: 1) selection of functional evidence-based synergistic drug combinations, validated in murine alveolar rhabdomyosarcoma (aRMS); 2) consensus modeling of multi-site drug sensitivity data to overcome intra-tumor heterogeneity, validated in epithelioid sarcoma (EPS); and 3) resistance abrogation by targeting of parallel biological pathways, validated in undifferentiated pleomorphic sarcoma (UPS).

## Methods

### Cell model establishment

The mouse primary tumor cell culture U23674 was established from a tumor at its site of origin in a genetically engineered *Myf6Cre,Pax3:Foxo1,p53* mouse bearing alveolar rhabdomyosarcoma (aRMS) as previously described [20]. In brief, the tumor was minced and digested with collagenase (10 mg/ml) overnight at 4 °C. Dissociated cells were then incubated in Dulbecco’s Modified Eagle’s Medium (DMEM) (11995–073; Thermo Fisher Scientific, Waltham, MA, USA) supplemented with 10% fetal bovine serum (FBS) (26,140,079; Thermo Fisher Scientific) and 1% penicillin-streptomycin (15140–122; Thermo Fisher Scientific) in 5% CO_2_ at 37 °C.

The human epithelioid sarcoma (EPS) sample PCB490 was collected from a patient undergoing planned surgical resection. Tumor tissue was partitioned into 5 distinct regions, minced and digested with collagenase type IV (10 mg/ml) overnight at 4 °C. The dissociated cells were then incubated in RPMI-1640 (11875–093; Thermo Fisher Scientific, Waltham, MA, USA) supplemented with 10% fetal bovine serum (FBS) and 1% penicillin-streptomycin in 5% CO_2_ at 37 °C. Sections 3, 4, and 5 (PCB490–3, PCB490–4, PCB490–5) successfully grew in culture. Samples from each region were also sent to The Jackson Laboratory (JAX) for patient-derived xenograft (PDX) model establishment. Cultures were maintained at low passage to minimize biological variation from the original patient tumor. Remaining tumor pieces were snap frozen for future DNA, RNA and protein isolation.

The human EPS sample PCB495 was received through the CCuRe-FAST tumor bank program. To create the cell cultures from the PCB495 primary tumor, the tumor was minced and digested with collagenase (10 mg/ml) overnight at 4 °C. The dissociated cells were then incubated in RPMI-1640 media supplemented with 10% fetal bovine serum (FBS) and 1% penicillin-streptomycin in 5% CO_2_ at 37 °C.

The human undifferentiated pleomorphic sarcoma (UPS) PCB197 was received through the CCuRe-FAST tumor bank program. To create the cell cultures from the PCB197 primary tumor, the tumor was minced and digested with collagenase (10 mg/ml) overnight at 4 °C. The dissociated cells were then incubated in RPMI-1640 media supplemented with 10% fetal bovine serum (FBS) and 1% penicillin-streptomycin in 5% CO_2_ at 37 °C.

All human tissue samples were acquired through the Childhood Cancer Registry for Familial and Sporadic Tumors (CCuRe-FAST) tumor banking program. All patients enrolled in CCuRe-FAST provided informed consent. All aspects of the study were reviewed and approved by the Oregon Health & Science University (OHSU) Institutional Review Board (IRB). Patient data and clinical and pathologic information are maintained in a de-identified database.

The canine UPS sample S1–12 was obtained from Oregon State University’s (OSU) College of Veterinary Medicine. OSU Institutional Animal Care and Use Committee (IACUC) approval was obtained for procurement of the tissue. To establish S1–12 cell culture, tumor tissue was minced and digested with collagenase (10 mg/ml) overnight at 4 °C. The dissociated cells were then incubated in RPMI-1640 media supplemented with 10% fetal bovine serum (FBS) and 1% penicillin-streptomycin in 5% CO_2_ at 37 °C.

### Immunoblotting of PCB490

Tumor tissue and cells from PCB490–1,2, and 5 were lysed in radioimmunoprecipitation (RIPA) buffer containing both protease and phosphatase inhibitors (Sigma Aldrich, St. Louis, MO). Lysates were homogenized and clarified by centrifugation at 14,000 rpm for 10 min. Thirty μg of protein was electrophoresed in 7.5% polyacrylamide gels, transferred to PVDF membranes for immunoblot analysis with mouse anti-BAF47 antibody (cat. 612,110, BD Biosciences, San Jose, CA) and mouse anti-β-actin antibody (cat. A1978, Sigma Aldrich), and developed by chemiluminescence (cat. 170–5061, BioRad Clarity Western ECL Substrate, Hercules, CA) per the manufacturer’s protocol.

### Cell lines

The VA-ES-BJ (Accession CVCL_1785) cell line was purchased commercially (cat# CRL-2138, ATCC, Manassas, VA). The cell line VA-ES-BJ has been validated before shipment by STR profile and mycoplasma testing. The cell line was used for the experiments directly after reception of the cell line.

The ESX cell line was provided by author TT [21]. The FU-EPS-1 (Accession CVCL_E311) cell line was provided by author JNishio [22].

Neither ESX nor FU-EPS-1 have available STR validation profiles, and so comparison to a standard STR profile cannot be performed. However, both cell lines were checked for INI1 loss consistent with EPS cell lines. Cell lines were tested for mycoplasma with the Lonza MycoAlert Plus test kit. Cat. LT07–703, Lonza Bioscience, Singapore).

### Patient derived xenograft (PDX) model development

All aspects of cancer tissue sharing for model development were reviewed and approved by the Oregon Health & Science University Institutional Review Board. The PCB490 PDX model was generated at JAX (model number J00007860) by implanting surgical human tumor tissue into 4–6-week-old female immunodeficient NOD.Cg-*Prkdc*^*scid*^ *Il2rg*^*tm1Wjl*^/SzJ (NSG) mice without prior in vitro culturing of the tumor cells. Time from surgery to implantation was approximately 24 h. Once a xenografted tumor reached ~ 1000 mm^3^, the tumor was harvested and divided into 3–5 mm^3^ fragments. Fragments were implanted into five 6–8-week-old female NSG mice for expansion to P1. Other fragments were sent for quality control assessment (see below). The remaining fragments were cryopreserved in 10% DMSO. When P1 tumors reached 1000mm^3^ they were harvested and divided into quarters: ¼ for quality control, ¼ snap frozen for genomics, ¼ placed into RNALater (Ambion) for RNA-seq, and the remaining ¼ divided into 3–5 mm^3^ pieces and cryopreserved in 10% DMSO.

The quality control procedures employed for PDX model development included testing the patient tumor for LCMV (lymphocytic choriomeningitis virus), bacterial contamination, and tumor cell content. The engrafted tumors at P0 and P1 were DNA fingerprinted using a Short Tandem Repeat (STR) assay to ensure model provenance in subsequent passages.

Model details available online at:

http://tumor.informatics.jax.org/mtbwi/pdxDetails.do?modelID=J000078604

Immunohistochemistry (IHC) for human CD45 (IR75161–2, Agilent Technologies) was performed on paraffin embedded blocks of engrafted tumors to identify cases of lymphomagenesis which have been reported previously in PDXs. IHC for human ki67 (IR62661–2, Agilent Technologies) was used to ensure the propagated tumors were human in origin. H&E sections of engrafted tumors were reviewed by a board-certified pathologist (RGE) to evaluate concordance of the morphological features of the engrafted tumor to the patient tumor. Further, tissue was stained with vimentin (IR63061–2, Agilent Technologies) to confirm human origin.

Model information is publicly accessible at: http://tumor.informatics.jax.org/mtbwi/pdxSearch.do

### Chemical screens

Four chemical screens were used to generate functional drug screening data. The first screen was a custom 60 agent chemical screen of well-characterized target inhibitors denoted the Pediatric Preclinical Testing Initiative Screen Version 2.1 (PPTI screen). Chemical concentrations of agents in all chemical screens were either [10 nM, 100 nM, 1 μM, 10 μM] or [100 nM, 1 μM, 10 μM, 100 μM] depending on compound activity range. Fifty-four of the 60 drugs on the chemical screen have a published quantified drug-target inhibition profile.

The second screen was a custom 60 agent chemical screen denoted Drug Screen V3 consisting of a variety of small molecule kinase inhibitors, epigenetic target inhibitors, and cell cycle inhibitors. Fifty-two of 60 drugs on the chemical screen have a published drug-target inhibition profile.

The third chemical screen was a GlaxoSmithKline open access Orphan Kinome-focused chemical screen (denoted GSK screen) consisting of 402 novel and newly characterized tool compounds [23] with target inhibition profiles quantified by Nanosyn Screening and Profiling Services. Drug-target interaction was assayed over 300 protein targets for each of the 402 compounds. The compounds were tested at 100 nM and 10 μM concentrations to bracket the drug-target EC_50_ values. The final EC_50_ values used for analysis of the chemical screen results were inferred from the available data using hill curve fitting to predict the 50% inhibition point.

The final screen was a Roche-developed open access chemical screen (denoted Roche screen) consisting of 223 novel kinase inhibitor compounds [24]. Roche screen compounds had a mixture of quantified or qualified drug-target inhibition profiles, though drug-target inhibition profiles were made available only for sensitive compounds.

Cell cultures were plated in 384-well plates at a seeding density of 5000 cells per well onto gradated concentrations of drug screen compounds. Cells were incubated in model-specific culture media at 37 °C, with 5% CO_2_, for 72 h. Cell viability was assessed by CellTiter-Glo® Luminescent Cell Viability Assay (cat. G7570, Promega, Madison, WI) per manufacturer’s protocol. Luminescence was measured using a BioTek Synergy HT plate reader (BioTek, Winooski, VT). Single agent IC_50_ values were determined using a hill curve-fitting algorithm with variable hill slope coefficients performed in Microsoft Excel. Manual curation and re-fitting of the results was performed before results were finalized.

U23674 primary tumor culture was assayed via three drug screens: PPTI drug screen, GSK drug screen, and the Roche drug screen (Additional files 1, 2, 3: Figures S1-S3 and Additional files 15, 16, 17, 18, 19, 20, 21: Tables S1-S7). S1–12 primary tumor culture was screened using the PPTI screen (Additional file 36: Table S22). PCB197 primary tumor culture was screened using the PPTI screen (Additional file 36: Table S22). PCB490–3, PCB490–4, PCB490–5 primary cultures were screened with Drug Screen V3 and the Roche drug screen (Fig. 3, Additional files 30, 31: Tables S15 and S16). Cell lines ESX, FU-EPS-1, and VA-ES-BJ were screened with Drug Screen V3 (Additional file 35: Table S21). PCB495 primary culture was screened with Drug Screen V3 (Additional file 35: Table S21).

### U23674 drug combination studies and calculation of combination index (CI)

U23674 drug combination validation experiments were guided by GlaxoSmithKline chemical screen PTIM models. Single agent validations to calculate independent drug efficacy were performed at dosages in the range of 5 nM to 100 μM to bracket IC_50_ and IC_25_ dosage values; for combination experiments, the IC_25_ dosage for one agent was tested in combination with gradated dosages (5 nM to 100 μM) of the complementary agent, and vice versa. Single agent and combination agent validation experiments were performed at passage 5.

CI values were generated using the CompuSyn software tool. Effect values for CompuSyn monotherapy and combination therapy were determined by mean cell death based on *n* = 3 technical replicates with *n* = 4 replicates for the following treatment conditions: OSI-906, GDC-0941, OSI-906 + GDC-0941 (OSI-906 at IC_25_ + GDC-0941 at varying dosage, OSI-906 at varying dosage + GDC-0941 at IC_25_). CompuSyn CI values were calculated using the non-constant combination setting [25] (Additional file 28: Table S14).

We performed low-dose validation experiments to verify PTIM-identified synergistic mechanisms of action; reduced dosages of the combination agents were set to 5 times the EC_50_ value for the predicted target (175 nM OSI-906, 50 nM GDC-0941). CompuSyn CI values to validate the mechanism of synergy were calculated using the non-constant combination setting [25] (Additional file 28: Table S14).

In both regular dose and low dose experiments, CI values are reported only for functionally relevant dosages, i.e. dosages between the drug target’s EC_50_ and the drug’s maximum achievable human clinical dosage (C_max_). For OSI-906, the functional range is approximately [10 nM, 5 μM] (mouse pharmacokinetics: ~ 16 μM C_max_, 6.16 μM C_ss_; human pharmacokinetics: ~ 1.481 μM C_max_, 720 nM C_ss_). For GDC-0941, the functional range is approximately [5 nM, 1 μM] (mouse pharmacokinetics: ~ 12 μM C_max_, 1.59 μM C_ss_, human pharmacokinetics: ~ 1.481 μM C_max_, 720 nM C_ss_). CI values outside these ranges are denoted as N/A in Additional file 28: Table S14.

### U23674 exome sequencing analysis

Somatic point mutations were identified using the Genome Analysis Toolkit [26] (GATK, version 3.5.0) from the Broad Institute. Captured DNA libraries were sequenced with the Illumina HiSeq 1000 in paired-end mode. The reads that passed the Illumina BaseCall chastity filter were used for subsequent analysis. The mate pairs were pooled and mapped as single reads to the NCBI GRCm38/mm10 reference genome using the Burrows-Wheeler Aligner [27] (version 0.7.12), with shorter split hits marked as secondary to ensure compatibility with downstream tools. Identified PCR duplicates, defined as reads likely originating from the same original DNA fragments, were removed using Picard Tools MarkDuplicates (version 1.133). Mapping artifacts introduced during initial mapping are realigned using the GATK IndelRealigner, and base quality score recalibration to empirically adjust quality scores for variant calling was performed by the GATK BaseRecalibrator tool. The same process was used to process both the tumor sample and the matched normal tail sample. Variant discovery was performed by MuTect2 [28], with the NCBI GRCm38/mm10 dbSNP database used to filter known polymorphisms present in the paired sample. Variant annotation and effect prediction was performed using SnpEff [29] using the GRCm38.81 database. Only medium and high impact effect variants are considered for the purpose of downstream analysis and reporting in figures. Exome analysis protocol is based on the GATK Best Practices protocol.

VarScan2 was used for copy number variation analysis of the paired tumor-normal data [30]. The Burrows-Wheeler Aligner was used to align the tumor and normal samples to NCBI GRCm38/mm10 reference genome as described previously. Samtools (version 0.1.19) mpileup tool with minimum mapping quality of 10 was used to generate the pileup file required by the VarScan2 copycaller function; log_2_ exon coverage ratio data from copycaller was segmented using DNAcopy with the undo.splits = “sdundo” parameter, and deviation from the null hypothesis set above 3 standard deviations. Genes in segments with segment mean above 0.25 or below − 0.25 and with *p*-value below 1e-10 were called as gained or lost, respectively. Copy number variation analysis protocol was partly based on the VarScan2 user manual [31].

### U23674 RNA deep sequencing analysis

RNA sequencing was performed on a low-passage U23674 culture, and on the control sample consisting of regenerating mouse muscle tissue following cardiotoxin injury in vivo. The paired-end raw reads were aligned to the NCBI GRCm38/mm10 reference mouse genome using TopHat version 2.0.9 [32] using Bowtie2 as the short-read aligner. Up to two alignment mismatches were permitted before a read alignment was discarded. The aligned reads were assembled into transcripts using Cufflinks version 2.1.1 [33]. Differential gene expression of tumor sample vs. control was performed by Cuffdiff using standard parameters. RNA analysis protocol was largely based on the approach described in the Tophat2 publication [34]. Quantified gene expression is provided in Additional file 23: Table S9.

### PCB490 exome sequencing analysis

Somatic point mutations were identified using the Genome Analysis Toolkit [26] (GATK, version 3.8.0) from the Broad Institute. Captured DNA libraries were sequenced in paired-end mode via the BGISeq 500 system at Beijing Genomics Institute. The reads that passed the Illumina BaseCall chastity filter were used for subsequent analysis. The mate pairs were pooled and mapped as single reads to the NCBI GRCh38 reference genome using the Burrows-Wheeler Aligner [27] (version 0.7.12), with shorter split hits marked as secondary to ensure compatibility with downstream tools. Identified PCR duplicates, defined as reads likely originating from the same original DNA fragments, were removed using Picard Tools MarkDuplicates (version 1.133). Mapping artifacts introduced during initial mapping are realigned using the GATK IndelRealigner, and base quality score recalibration to empirically adjust quality scores for variant calling was performed by the GATK BaseRecalibrator tool. The same process was used to process both the tumor sample and the matched normal sample. Variant discovery was performed by MuTect2 [28], with the NCBI GRCh38 dbSNP database used to filter known polymorphisms present in the paired sample. Variant annotation and effect prediction was performed using SnpEff [29] using the GRCh38.87 database. Only medium and high impact variants are considered for the purpose of downstream analysis and reporting in figures. Exome analysis protocol is based on the GATK Best Practices protocol.

VarScan2 was used for copy number variation analysis of the paired tumor-normal data [30]. The Burrows-Wheeler Aligner was used to align the tumor and normal samples to NCBI GRCh38 reference genome as described previously. Samtools (version 1.6) mpileup tool with minimum mapping quality of 10 was used to generate the pileup file required by the VarScan2 copycaller function; log_2_ exon coverage ratio data from copycaller was segmented using DNAcopy with the undo.splits = “sdundo” parameter, and deviation from the null hypothesis set above 3 standard deviations. Genes in segments with segment mean 2 standard deviations above or below ±0.5 and with *p*-value below 1e-10 were called as gained or lost, respectively. Copy number variation analysis protocol was partly based on the VarScan2 user manual [31].

### PCB490 RNA deep sequencing analysis

The PCB490 transcriptome library was sequenced with the Illumina HiSeq 2500 in paired-end mode. The reads that passed the chastity filter of Illumina BaseCall software were used for subsequent analysis. The paired-end raw reads for each RNA-seq sample were aligned to the UCSC hg38 reference human genome using Bowtie2 as the short-read aligner [32] using, allowing up two alignment mismatches before a read alignment was discarded. The aligned reads were assembled into transcripts using Cufflinks version 2.1.1 [33] and quantification was performed with Cuffquant [33]. RNA analysis protocol was adapted from the approach described in the original TopHat2 publication [34] (Additional file 33: Table S19).

### RAPID siRNA screen of U23674

U23674 underwent functional single gene knockdown (siRNA interference screen, Additional file 24: Table S10), however siRNA results were inconsistent with drug screening data (Additional file 25: Table S11) and are thus relegated to the supplement.

To assess the contribution of individual receptor tyrosine kinases to survival of U23674, we performed RAPID siRNA knockdown screening of U23674. Efficacy of single target knockdown of 85 members of the mouse tyrosine kinase family was performed as previously described [35]. Target sensitivity was determined by resulting cell viability quantified using an MTT assay (M6494; Thermo Fisher Scientific, Waltham, MA, USA). Targets with viability two standard deviations below the mean were identified as high-importance targets [35] (Additional file 24: Table S10).

### Phosphoproteomic screen of U23674

U23674 underwent phosphoproteome quantification (Kinexus phosphoproteomics analysis, Additional file 26: Table S12), however phosphoproteomics results were inconsistent among sample replicates and are thus relegated to the supplement.

To identify differentially phosphorylated protein targets, phosphoproteomics assays (Kinexus, Vancouver, British Columbia, Canada) were used to compare two duplicate cell lysates from U23674 against two duplicate cell lysates from regenerating muscle tissue acting as normal control. To perform the phosphoproteomics analyses, 50 μg of protein lysate from each sample was covalently labeled with a proprietary fluorescent dye. Free dye molecules were removed by gel filtration. After blocking non-specific binding sites on the array, an incubation chamber was mounted onto the microarray to permit the loading of related samples side by side on the same chip. Following sample incubation, unbound proteins were washed away. Each array produces a pair of 16-bit images, which are captured with a Perkin-Elmer ScanArray Reader laser array scanner. Signal quantification was performed with *ImaGene 8.0* from BioDiscovery with predetermined settings for spot segmentation and background correction. The background-corrected raw intensity data are logarithmically transformed. Z scores are calculated by subtracting the overall average intensity of all spots within a sample from the raw intensity for each spot, and dividing it by the standard deviations (SD) of all of the measured intensities within each sample (Additional file 26: Table S12).

### Probabilistic target inhibition maps

The Probabilistic Target Inhibition Map (PTIM) approach considers that the underlying mechanism for sensitivity to targeted drugs can be represented by a combination of parallel target groups (all parallel targets need to be inhibited to slow or stop tumor proliferation, similar to Boolean ‘AND’ logic) and series target groups (inhibiting any all targets in any target group will slow or stop tumor proliferation, similar to Boolean ‘OR’ logic). For estimating the series and parallel targets, we analyze cancer cell response to multi-target single agent drugs with overlapping but distinct target sets. For instance, drugs having the same selective target (such as pelitinib and erlotinib, which are potent inhibitors of the kinase target *EGFR*) can show different sensitivity in vitro which can be attributed to the biologically relevant side targets of the drugs. Our framework considers primary and secondary drug targets and generates logical groupings of targets (as single-target or multi-target blocks) that best explain chemical screen response data. We now incorporate secondary information to refine PTIM models.

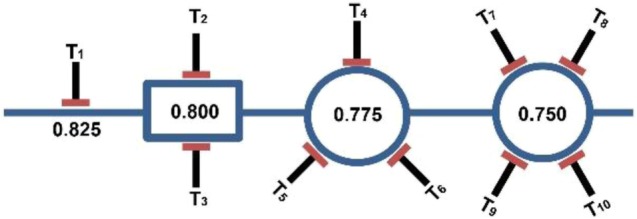



#### PTIM circuit models

PTIM models are visually represented as circuit models. Each “block” in the circuit represents a combination of two or more gene targets that explain sensitivity of a set of single agent compounds. The drug set represented by an individual block is determined by the PTIM objective function and feature selection algorithm [14, 16], and depends on the biological data inputs to the PTIM algorithm.

PTIM circuits consist of multiple blocks. Generally, only target combinations of one to four targets are considered during PTIM modeling. Blocks of one target (represented as single inhibitor symbol, T_1_) are called “single points of failure”, i.e. single targets which alone explain the sensitivity of one or more drug screen agents. Combinations of two targets are visually represented by a rectangular block with two inhibitor symbols (block T_2_ – T_3_). Combinations of three targets are visually represented by a circular block with three inhibitor symbols (block T_4_ – T_5_ – T_6_). Combinations of four targets are visually represented by a circular block with four inhibitor symbols (block T_7_ – T_8_ – T_9_ – T_10_). Each block has an associated score value (e.g. 0.825, 0.800, 0.775, 0.750, respectively) that represents the scaled sensitivity of all drug screen agents grouped in the block’s target combination [14, 16]. In brief, all single agent sensitivities (as IC_50_ values) are log_10_ scaled and converted to [0,1] sensitivity values via the following equation:

$$ \mathrm{sensitivity}=\left\{\begin{array}{c}\ \\ {}\frac{\log \left(\mathrm{maxTestedDose}\right)-\log \left({\mathrm{IC}}_{50}\right)}{\log \left(\mathrm{maxTestedDose}\right)},{\mathrm{IC}}_{50}>{\mathrm{C}}_{\mathrm{max}}\\ {}1,{\mathrm{IC}}_{50}\le {\mathrm{C}}_{\mathrm{max}}\ \end{array}\right. $$

Thus, the lower the IC_50_, the higher the sensitivity score. The score assigned to each block is a determined by the sensitivity of the drug screen agents assigned to the block following several correction factors [14, 16]. The shape of blocks in PTIM circuits are meant to serve as a convenient visual representation; ordering of PTIM circuit blocks are determined by overall score, with highest scored blocks on the left descending to lowest scored blocks on the right. The general PTIM algorithm is presented in previously published work [14, 16–18]. Methods for integration of secondary biological data are provided in the methods sections for modeling of U23674 and modeling of PCB490.

#### Synergy, heterogeneity, and resistance via PTIM models

PTIM circuit models are also designed to visually represent the clinical challenges PTIM modeling seeks to address. Synergistic drug combinations can be selected for any block with two or more targets by selecting two (or more) drugs which inhibit all targets in the block; the selected combination should kill cancer cells while monotherapy treatment would not. For example, based on (block T_2_ – T_3_), a drug inhibiting T_2_ and a drug inhibiting T_3_ will individually not slow tumor growth for the sample patient, while the combination T_2_ + T_3_ will.

Drug screening multiple spatially-distinct sites from a solid tumor can result in heterogeneous single agent sensitivity. Target group blocks identified as common amongst PTIM models from each distinct region can be used to design a drug combination that should slow or stop tumor growth across the entire heterogeneous tumor. Multi-site PTIM models can thus define heterogeneity-aware drug combinations.

Each block in a PTIM circuit represents a set of effective treatment options; effective options on parallel biological pathways represent multiple distinct treatment options which can individually slow tumor growth. A drug combination which inhibits multiple parallel biological pathway blocks can shut down potential survival mechanisms for cancer cells, thus abrogating development of resistance. Series PTIM blocks can thus define resistance abrogating drug combinations.

### Integrative nonlinear Boolean modeling for U23674

Probabilistic Target Inhibition Maps (PTIMs) were used for integrative analysis of U23674 biological data [16–18].

#### RNA-seq integration

For targets common to both RNA expression data and drug-target interaction data, we use gene expression data to eliminate possible false positives from chemical screen results and to narrow down the true positives among relevant targets identified by the PTIM approach. False positives are defined here as targets that are inhibited by effective drugs but are not expressed in cancer cells at levels above matched normal cells. Note that we consider the effect of a molecularly-targeted drug is to inhibit the target when it is expressed, thus under-expressed drug targets will have limited impact on drug response. Here, over-expression is determined as gene expression in the tumor sample 50% greater than that in the control sample. The RNA-seq target set is used for PTIM creation via the published model development algorithms.

Formally, RNA-seq data is integrated as below:

$$ \mathrm{T}:= \mathrm{targets}\ \mathrm{in}\mathrm{hibited}\ \mathrm{in}\ \mathrm{drug}\ \mathrm{screen} $$

$$ \mathrm{G}:= \mathrm{targets}\ \mathrm{with}\ \mathrm{RNA}-\mathrm{seq}\ \mathrm{expression}\ \mathrm{in}\ \mathrm{tumor}\ \mathrm{and}\ \mathrm{normal}\ \mathrm{cells} $$

$$ \mathrm{Tumor}\left(\mathrm{x}\right):= \mathrm{gene}\ \mathrm{expression}\ \mathrm{of}\ \mathrm{target}\ \mathrm{x}\ \mathrm{in}\ \mathrm{tumor}\ \mathrm{sample} $$

$$ \mathrm{Normal}\left(\mathrm{x}\right):= \mathrm{gene}\ \mathrm{expression}\ \mathrm{of}\ \mathrm{target}\ \mathrm{x}\ \mathrm{in}\ \mathrm{normal}\ \mathrm{sample} $$

$$ {\forall}_{\mathrm{x}\in \mathrm{T}\cap \mathrm{G}}\ \mathrm{Ratio}\left(\mathrm{x}\right):= \mathrm{T}\mathrm{umor}\left(\mathrm{x}\right)/\mathrm{Normal}\left(\mathrm{x}\right) $$

$$ {\forall}_{\mathrm{x}\in \mathrm{T}\cap \mathrm{G}}\ \left\{\begin{array}{c}\mathrm{if}\ \mathrm{Ratio}\left(\mathrm{x}\right)\ge 1.5,\mathrm{keep}\ \mathrm{target}\ \mathrm{x}\ \mathrm{for}\ \mathrm{consideration}\ \\ {}\mathrm{if}\ \mathrm{Ratio}\left(\mathrm{x}\right)<1.5,\mathrm{remove}\ \mathrm{target}\ \mathrm{x}\ \mathrm{from}\ \mathrm{consideration}\end{array}\right. $$

$$ {\forall}_{\mathrm{x}\notin \mathrm{T}\cap \mathrm{G}}\ \mathrm{keep}\ \mathrm{target}\ \mathrm{x}\ \mathrm{for}\ \mathrm{consideration} $$

#### Exome-seq integration

We use exome sequencing data to identify targets likely important in the biological function of tumor cells. We assume that genetic variants may explain the behavior of compounds inhibiting the mutated/altered targets. Depending on the available evidence for mutations and variations, targets are incorporated into the model search or final PTIM model via the published model development algorithms.

Formally, exome-seq data is integrated as below:

$$ \mathrm{T}:= \mathrm{targets}\ \mathrm{in}\mathrm{hibited}\ \mathrm{in}\ \mathrm{drug}\ \mathrm{screen} $$

$$ \mathrm{G}:= \mathrm{targets}\ \mathrm{with}\ \mathrm{RNA}-\mathrm{seq}\ \mathrm{expression}\ \mathrm{in}\ \mathrm{tumor}\ \mathrm{and}\ \mathrm{normal}\ \mathrm{cells} $$

$$ \mathrm{Mut}\left(\mathrm{x}\right):= \mathrm{mutation}/\mathrm{indel}\ \mathrm{status}\ \mathrm{of}\ \mathrm{target}\ \mathrm{x}\ \left(\mathrm{low}/\mathrm{med}/\mathrm{high}\ \mathrm{impact}\ \mathrm{mutation}/\mathrm{indel}\right) $$

$$ \mathrm{CNV}\left(\mathrm{x}\right):= \mathrm{copy}\ \mathrm{number}\ \mathrm{status}\ \mathrm{of}\ \mathrm{target}\ \mathrm{x}\ \left(\mathrm{gain}/\mathrm{loss}\right) $$

$$ {\forall}_{\mathrm{x}\in \mathrm{T}\cap \mathrm{G}}\left\{\begin{array}{c}\ \\ {}\mathrm{if}\ \mathrm{Mut}\left(\mathrm{x}\right)=\mathrm{high}\ \mathrm{AND}\ \mathrm{CNV}\left(\mathrm{x}\right)=\mathrm{gain},\mathrm{include}\ \mathrm{target}\ \mathrm{x}\ \mathrm{in}\ \mathrm{model}\ \\ {}\mathrm{if}\ \mathrm{Mul}\left(\mathrm{x}\right)=\mathrm{med}\ \mathrm{AND}\ \mathrm{CNV}\left(\mathrm{x}\right)=\mathrm{gain},\mathrm{add}\ \mathrm{target}\ \mathrm{x}\ \mathrm{to}\ \mathrm{initial}\ \mathrm{search}\ \mathrm{conditions}\ \\ {}\mathrm{if}\ \mathrm{Mut}\left(\mathrm{x}\right)=\mathrm{high},\mathrm{add}\ \mathrm{target}\ \mathrm{x}\ \mathrm{to}\ \mathrm{initial}\ \mathrm{search}\ \mathrm{conditions}\ \\ {}\mathrm{if}\ \mathrm{Mut}\left(\mathrm{x}\right)=\mathrm{med}\ \mathrm{OR}\ \mathrm{CNV}\left(\mathrm{x}\right)=\mathrm{gain},\mathrm{keep}\ \mathrm{target}\ \mathrm{x}\ \mathrm{in}\ \mathrm{model}\ \mathrm{once}\ \mathrm{added}\ \\ {}\mathrm{otherwise},\mathrm{do}\ \mathrm{nothing}\ \end{array}\right. $$

$$ {\forall}_{\mathrm{x}\notin \mathrm{T}\cap \mathrm{G}}\ \mathrm{keep}\ \mathrm{target}\ \mathrm{x}\ \mathrm{for}\ \mathrm{consideration} $$

#### RAPID siRNA screen integration

RAPID screen results identify high sensitivity single target mechanisms of cancer cell growth inhibition; identified hit targets were set as “required” (forced inclusion) in the RAPID siRNA PTIM model effective as sensitive siRNA targets may explain drug sensitivity of agents inhibiting the siRNA targets. Targets not identified by RAPID screening could still have effect in multi-target combinations, and thus were retained for consideration. The RAPID target set is used for PTIM creation via the published model development algorithms.

Formally, RAPID siRNA data is integrated as below:

$$ \mathrm{T}:= \mathrm{targets}\ \mathrm{in}\mathrm{hibited}\ \mathrm{in}\ \mathrm{drug}\ \mathrm{screen} $$

$$ \mathrm{G}:= \mathrm{targets}\ \mathrm{with}\ \mathrm{RAPID}\ \mathrm{siRNA}\ \mathrm{viability}\ \mathrm{data} $$

$$ \mathrm{RAPID}\left(\mathrm{x}\right):= \mathrm{cell}\ \mathrm{viability}\ \mathrm{following}\ \mathrm{siRNA}\ \mathrm{knockdown}\ \mathrm{of}\ \mathrm{target}\ \mathrm{x} $$

$$ \left(\upmu, \upsigma \right):= \mathrm{mean}\ \mathrm{and}\ \mathrm{standard}\ \mathrm{deviation}\ \mathrm{of}\ \mathrm{RAPID}\ \mathrm{siRNA}\ \mathrm{dataset} $$

$$ {\forall}_{\mathrm{x}\in \mathrm{T}\cap \mathrm{G}}\ \left\{\begin{array}{c}\mathrm{if}\ \mathrm{RAPID}\left(\mathrm{x}\right)+2\upsigma <1.5,\mathrm{add}\ \mathrm{target}\ \mathrm{x}\ \mathrm{to}\ \mathrm{PTIM}\ \mathrm{model}\\ {}\mathrm{if}\ \mathrm{RAPID}\left(\mathrm{x}\right)+2\upsigma \ge 1.5,\mathrm{nothing}\ \end{array}\right. $$

$$ {\forall}_{\mathrm{x}\notin \mathrm{T}\cap \mathrm{G}}\ \mathrm{keep}\ \mathrm{target}\ \mathrm{x}\ \mathrm{for}\ \mathrm{consideration} $$

#### Kinexus phosphoproteomics screen integration

The phosphoproteomics screen results identify differentially phosphorylated targets and associated pathways, phosphorylation of these targets may be pushing the system towards a particular phenotype, and intervention in the form of changing phosphorylation status might result in significant changes to the system. Targets identified as overactive in tumor compared to normal are included in the target set for the PTIM model. The phosphoproteomics target set is used for PTIM creation via the published model development algorithms.

$$ \mathrm{T}:= \mathrm{targets}\ \mathrm{in}\mathrm{hibited}\ \mathrm{in}\ \mathrm{drug}\ \mathrm{screen} $$

$$ \mathrm{G}:= \mathrm{targets}\ \mathrm{with}\ \mathrm{RAPID}\ \mathrm{siRNA}\ \mathrm{viability}\ \mathrm{data} $$

$$ {\mathrm{P}}_1\left(\mathrm{x}\right):= \mathrm{z}-\mathrm{score}\ \mathrm{ratio}\ \mathrm{of}\ \mathrm{target}\ \mathrm{x}\ \mathrm{in}\ \mathrm{U}23674\ \mathrm{replicate}\ 1\ \mathrm{vs}\ \mathrm{normal} $$

$$ {\mathrm{P}}_2\left(\mathrm{x}\right):= \mathrm{z}-\mathrm{score}\ \mathrm{ratio}\ \mathrm{of}\ \mathrm{target}\ \mathrm{x}\ \mathrm{in}\ \mathrm{U}23674\ \mathrm{replicate}\ 2\ \mathrm{vs}\ \mathrm{normal} $$

$$ {\forall}_{\mathrm{x}\in \mathrm{T}\cap \mathrm{G}}\ \left\{\begin{array}{c}\mathrm{if}\ \left[\ {\mathrm{P}}_1\left(\mathrm{x}\right)\ge 1\ \mathrm{and}\ |{\mathrm{P}}_1\left(\mathrm{x}\right)-{\mathrm{P}}_2\left(\mathrm{x}\right)|\le 0.5\ \right],\mathrm{add}\ \mathrm{target}\ \mathrm{x}\ \mathrm{to}\ \mathrm{PTIM}\ \mathrm{model}\ \\ {}\mathrm{if}\ \left[\ {\mathrm{P}}_1\left(\mathrm{x}\right)\ge 1\ \mathrm{and}\ |{\mathrm{P}}_1\left(\mathrm{x}\right)-{\mathrm{P}}_2\left(\mathrm{x}\right)|>0.5\ \right],\mathrm{nothing}\ \end{array}\right. $$

$$ {\forall}_{\mathrm{x}\notin \mathrm{T}\cap \mathrm{G}}\ \mathrm{keep}\ \mathrm{target}\ \mathrm{x}\ \mathrm{for}\ \mathrm{consideration} $$

### Integrative nonlinear Boolean modeling for PCB490

Probabilistic Target Inhibition Maps (PTIMs) were used for integrative analysis of heterogeneous PCB490 biological data [16–18].

#### RNA-seq integration

RNA sequencing data for PCB490–5 was used to eliminate under-expressed targets from consideration for PTIM model development, reducing the potential number of models. Due to possessing only tumor tissue for PCB490, RNA sequencing was performed only on the tumor sample; targets with quantified expression above the first quantile were retained for PTIM model development. The RNA-seq target set is used for PTIM creation via the published model development algorithms.

Formally, RNA-seq data is integrated as below:

$$ \mathrm{T}:= \mathrm{targets}\ \mathrm{in}\mathrm{hibited}\ \mathrm{in}\ \mathrm{drug}\ \mathrm{screen} $$

$$ \mathrm{G}:= \mathrm{targets}\ \mathrm{with}\ \mathrm{RNA}-\mathrm{seq}\ \mathrm{expression}\ \mathrm{in}\ \mathrm{tumor}\ \mathrm{and}\ \mathrm{normal}\ \mathrm{cells} $$

$$ \mathrm{Tumor}\left(\mathrm{x}\right):= \mathrm{gene}\ \mathrm{expression}\ \mathrm{of}\ \mathrm{target}\ \mathrm{x}\ \mathrm{in}\ \mathrm{tumor}\ \mathrm{sample} $$

$$ {\mathrm{Q}}_1:= \mathrm{first}\ \mathrm{quartile}\ \mathrm{of}\ \mathrm{Tumor}\left(\ast \right)\ \mathrm{data} $$

$$ {\forall}_{\mathrm{x}\in \mathrm{T}\cap \mathrm{G}}\ \left\{\begin{array}{c}\mathrm{if}\ \mathrm{T}\mathrm{umor}\left(\mathrm{x}\right)\ge {\mathrm{Q}}_1,\mathrm{keep}\ \mathrm{target}\ \mathrm{x}\ \mathrm{for}\ \mathrm{consideration}\ \\ {}\mathrm{if}\ \mathrm{T}\mathrm{umor}\left(\mathrm{x}\right)<{\mathrm{Q}}_1,\mathrm{remove}\ \mathrm{target}\ \mathrm{x}\ \mathrm{from}\ \mathrm{consideration}\ \end{array}\right. $$

$$ {\forall}_{\mathrm{x}\notin \mathrm{T}\cap \mathrm{G}}\ \mathrm{keep}\ \mathrm{target}\ \mathrm{x}\ \mathrm{for}\ \mathrm{consideration} $$

#### PTIM Ensemble combination optimization

To address tumor heterogeneity concerns, PTIM computational models were generated for each of the drug screened PCB490 cultures (PCB490–3, PCB490–4, and PCB490–5). The PCB490–5 PTIM model integrates RNA sequencing data as above. Combination therapy for PCB490 was designed by identifying PTIM target blocks in each of the three different cell models druggable by the same two-drug combination.

### Rewiring experiments for U23674

Untreated U23674 cells were screened using the Roche Orphan Kinome Screen and concurrently used to establish 6 additional independent cultures grown in culture media at 37 °C with 5% CO2. Upon reaching 70% confluence, low dosage single agents and drug combinations (DMSO vehicle, 175 nM OSI-906, 50 nM GDC-0941, 175 nM OSI-906 + 50 nM GDC-0941) were added to culture plates and incubated for 72 h (Additional file 10: Figure S10). Cell plates were then washed in Phosphate Buffered Saline (PBS, Gibco, Grand Island, New York), trypsonized with Trypsin-EDTA (0.25%) (25,200,056, Thermo Fisher Scientific), and screened using the Roche Orphan Kinome Screen (Additional file 11: Figure S11, Additional file 29: Table S15). Rewiring data was used to generate PTIM models to identify post-intervention changes to U23674 survival pathways (Additional file 12: Figure S12, Additional file 27: Table S13).

### Resistance abrogation experiments for PCB197 and S1–12

PCB197 PPTI screen data and S1–12 PPTI screen data were used to generate PTIM models to identify canine and human cross-species mechanistic targets for undifferentiated pleomorphic sarcoma. Consensus targets were chosen for their appearance in human and canine PTIM models; two drugs (obatoclax, an MCL1 inhibitor and panobinostat, a pan-HDAC inhibitor) that most effectively inhibited PTIM-identified blocks at clinically achievable concentrations were selected for validation.

Potential for resistance abrogation by targeting 2 blocks common to both human and canine PTIM models directed a six-arm proof-of-principle experiment to show that inhibition of multiple blocks inhibited could abrogate tumor cell resistance. PCB197 and S1–12 cell cultures were seeded in quadruplicate on 6-well plates (6 plates per cell model) with 10,000 cells per well. Cells were plated 24 h prior to incubation with any drug. The drug concentrations chosen were 1.5 times the EC_50_ of the PTIM target of interest. The drug selection was based on desired targets, as well as requiring drug concentration for reaching 1.5 times target K_d_ must also be less than the maximum clinically achievable concentration.

One plate per cell model was assigned to each of the 6 treatment arms: 1) vehicle control; 2) obatoclax for 6 days; 3) panobinostat for 6 days; 4) obatoclax for 3 days, wash, then panobinostat for 3 days; 5) panobinostat for 3 days, wash, then obatoclax for 3 days; 6) obatoclax + panobinostat simultaneously for 6 days. After 6 days, culture plates were washed with PBS and fresh DMEM with 10% FBS was placed in each well. Wells were monitored until confluency was observed. The primary study endpoint was days to well confluency as determined by a single user. Cells were also counted manually with a hemocytometer and photographed to confirm consistency of the user’s definition of confluency. If after 100 days the cells did not reach confluency, the remaining cells are counted and the study concluded. The experimental design and results are available in Fig. 5**.**

### Orthotopic allograft studies for U23674

We orthotopically engrafted adult *SHO (*SCID/hairless/outbred) mice (Charles River, Wilmington, Massachusetts) with 10^6^ U23674 cells. Engraftment was performed after injuring the right gastrocnemius muscle by cardiotoxin injection as previously described [35]. Mice were assigned to treatment arms randomly without a specific assignment strategy. Treatment commenced 2 days after engraftment; mice were treated with vehicle control (tartaric acid + TWEEN80/methylcellulose), 50 mg/kg OSI-906, 150 mg/kg GDC-0941, and combination 50 mg/kg OSI-906 plus 150 mg/kg GDC-0941. Each arm was assigned *n* = 8 mice per arm. Sample size was selected to provide 90% power for the statistical tests. The GDC-0941 arm lost one mouse during oral gavage; the corresponding data point was censored. Dosing schedule was once daily by oral gavage up to day 5, at which time dosing was performed every other day due to weight loss on day 4. The change in dosing schedule stabilized weight loss. The endpoint considered for the study and survival analysis was tumor volume = 1.4 cc. All drug studies in mice were performed after receiving approval from the IACUC at Oregon Health and Science University. Variances between compared groups were similar per Greenwood’s Formula. No blinding was performed during in vivo experiments. No adverse events were noted. All animal procedures were conducted in accordance with the Guidelines for the Care and Use of Laboratory Animals and were approved by the Institutional Animal Care and Use Committee at the Oregon Health & Science University. At conclusion of the study, mice were sacrificed via isoflurane overdose followed by cervical dislocation.

### Patient derived xenograft (PDX) model testing for PCB490

Adult female stock mice (Envigo *Foxn1*^*nu*^ Athymic nudes) were implanted bilaterally with approximately 5x5x5mm fragments subcutaneously in the left and right flanks with JAX PDX model of Human Epithelioid Sarcoma (J000078604 (PCB490) – JAX-001). After the tumors reached 1–1.5 cm^3^, they were harvested and the viable tumor fragments approximately 5x5x5 mm were implanted subcutaneously in the left flank of the female study mice (Envigo *Foxn1*^*nu*^ Athymic nudes). Each animal was implanted with a specific passage lot and documented. J000078604 (PCB490) – JAX-001) was P4. Tumor growth was monitored twice a week using digital calipers and the tumor volume (TV) was calculated using the formula (0.52 × [length × width^2^]). When the TV reached approximately 150–250 mm^3^ animals were matched by tumor size and assigned into control or treatment groups (3/group for J000078604 (PCB490) – JAX-001). Dosing was initiated on Day 0. After the initiation of dosing, animals were weighed using a digital scale and TV was measured twice per week. For J000078604 (PCB490) – JAX-001, sunitinib (reconstituted in 5% DMSO + corn oil) was administered PO QD for 21 days at 30.0 mg/kg/dose and BEZ235 (reconstituted in 10% N-Methyl-2-pyrrolidone [NMP] + 90% polyethylene glycol 300) was administered PO QD for 21 days at 25.0 mg/kg/dose alone and in combination. No adverse events were noted. At conclusion of the study, mice were sacrificed via isoflurane overdose followed by cervical dislocation.

### Statistics

Spearman correlation coefficients for Epithelioid sarcoma drug screen response data were calculated in SAS, correlating drug screen IC_50_ values between all samples. Statistical comparison of correlation coefficients between separate groups was performed in SAS using two-tailed student’s T-test.

The Kaplan-Meier curves for the U23674 in vivo orthotropic allograft studies were generated and compared with logrank statistical tests. No blinding was performed. Analysis was performed by an external group of statisticians (MWG, BH, JM, SG).

*P*-values for the PCB490 PDX experiment were generated using a repeated measures linear model of tumor size in terms of group, time, and the group by time interaction based on an autoregressive order 1 correlation assumption with SAS Version 9.4 for Windows (SAS Institute, Cary, NC). Analysis was performed by an external group of statisticians (MWG, BH, JM).

## Results

### Computational analysis of functional and molecular data via PTIM analysis

The key PTIM modeling assumption is that in vitro drug sensitivity in cancer cells is driven by a small subset of key gene targets uniquely determined by the patient’s biology, and that patient-specific drug sensitivity is most accurately predicted by multivariate modeling of autologous drug sensitivity data. The PTIM pipeline requires drug screening data from multiple (60+) monotherapy agents with quantified drug-target EC_50_ values (Fig. 1, Testing Step). PTIM modeling specifically takes advantage of the promiscuity of targeted compounds by incorporating main-target and off-target EC_50_ values during modeling. Correspondingly, PTIM models will better represent the underlying biology of individual cancer samples when complete drug-target interaction EC_50_ information is available. Integration of additional patient-specific molecular data (e.g., exome-seq, RNA-seq, phosphoproteomics, siRNA-mediated gene knockdown, Fig. 1, Testing Step) identifies targets of interest to further refine target selection during model creation.

Drug sensitivity data and secondary molecular data are provided as inputs to the PTIM computational framework [14–19], which provides as output a mathematical model quantifying expected sensitivity of multi-target inhibition of the patient’s cancer cells. The model approaches sensitivity prediction as a feature selection machine learning problem, where the “features” are the gene targets inhibited by individual drugs. The objective of the PTIM analysis approach is to find feature sets which group sensitive and insensitive drugs together into binary “bins”, representing a set of inhibited targets. A feature set where drugs in the same bin have similar sensitivity values is considered more optimal than a feature set where bins have high variance. The addition of molecular sequencing data can eliminate certain features from consideration if they are absent in the tumor (e.g. no expression of the gene per RNA-seq data) or can increase likelihood of a feature being included in the model if the feature is of high interest (e.g. highly expressed per RNA-seq, or mutated per exome-seq). The full details of integration of molecular is available in the methods section, including a detailed description of integration of molecular data to drug screening data for validation experiments presented in this manuscript.

Multi-target sensitivity mechanisms are represented graphically as “tumor cell survival circuits” (Fig. 1, Modeling Step) where target combinations are denoted as “blocks” (e.g. Figure 1, Modeling Step inhibitor symbols A, B, C + D). The value in the center of each PTIM block represents expected scaled sensitivity following inhibition of associated block targets. The resulting PTIM model enables combination therapy assignment via matching of targets in high-sensitivity PTIM blocks to drugs in clinical investigation or clinical use. A single block denotes monotherapy (e.g. A, B) or combination therapy (synergistic targets, e.g. C + D), while multiple blocks represent independent treatments which can be leveraged to abrogate cancer cell resistance.

If PTIM models from spatially-distinct tumor sites are available, consensus therapy can be selected from distinct models to mitigate potential intra-tumor heterogeneity. When available, additional patient tumor tissue can be used to validate PTIM-predicted combination therapy in vitro or in vivo (Fig. 1, Validation Step). PTIM modeling is the foundation of our personalized therapy pipeline built with the goal to address the unmet clinical needs of the 600,000 patients dying from cancer every year [1].

The MATLAB package to generate basic PTIM models was published in conjunction with a previous publication [16] and is available online (http://www.myweb.ttu.edu/rpal/Softwares/PTIM1.zip).

### Proof-of-concept of synergy prediction by PTIM modeling

#### Chemical screening, biological interrogation, and PTIM modeling of a genetically engineered mouse model (GEMM)-origin aRMS

For our 2-drug synergy proof-of-concept study, we used a low passage primary tumor cell culture of a GEMM-origin aRMS tumor designated U23674 [36] as a pilot study of the PTIM personalized therapy pipeline. From our previous work [35, 37] we reasoned that kinases would be fundamental to the biology of aRMS, thus we interrogated U23674 drug sensitivity via three kinase inhibitor compound libraries: the GlaxoSmithKline (GSK) Open Science Orphan Kinome Library (GSK screen), the Roche Orphan Kinome Screen Library (Roche screen), and a custom Pediatric Preclinical Testing Initiative Drug Screen Version 2.1 (PPTI screen).

The GSK screen [38] consists of 305 compounds with experimentally quantified drug-target interaction EC_50_ values. Of the 305 screened compounds, 40 (13%) caused at least 50% cell growth inhibition at or below maximum tested in vitro dosage in U23674, hereafter defined as a compound “hit” (Additional file 1: Figure S1 and Additional files 15 and 16: Tables S1 and S2). The Roche screen consists of 223 novel kinase inhibitor compounds, most with quantified drug-target interactions; 21 of 223 compounds (9.4%) were hits on U23674 (Additional file 2: Figure S2 and Additional files 17, 18 and 19: Tables S3, S4 and S5). The PPTI screen consists of 60 preclinical- or clinical-stage targeted agents; 28 of 60 compounds (46.7%) were hits on U23674 (Additional file 3: Figure S3 and Additional files 20 and 21: Tables S6 and S7).

Additionally, U23674 primary tissue was sequenced to enhance therapy design (tumor whole exome sequencing, matched normal whole exome sequencing, and whole transcriptome sequencing, Additional files 22 and 23: Tables S8 and S9). Exome sequencing of U23674 did not identify any druggable targets both mutated and amplified (Additional file 4: Figure S4 and Additional files 22 and 23: Tables S8 and S9); six genes possessed activating mutations (*Fat4, Gm156, Mtmr14, Pcdhb8, Trpm7, Ttn, Zfp58*) and one gene possessed a high-impact frameshift indel (*Ppp2r5a*); none of these seven gene targets are druggable. No gene with a mutation or indel is druggable. Four druggable gene targets show evidence of copy number gain (*Gsk3a, Epha7, Psmb8, Tlk2*). *Gsk3a*, *Psmb8*, and *Tlk2* all show neutral expression or underexpression by RNA-seq. *Gsk3a* inhibitors were effective in 12 of 72 inhibitors (16.667%) across three screens, suggesting Gsk3a is not critical for cancer cell survival in U23674. *Psmb8* inhibition showed in vitro efficacy in nearly all tested cell cultures across multiple tumor types (unpublished internal data) and, along with lack of overexpression, was thus treated as an in vitro screening artifact; furthermore, clinical response of solid tumors to proteasome inhibitors has been limited [39]. *Tlk2* has no published inhibitor compounds. While overexpressed, the *Epha7* inhibitor on the PPTI drug screen was ineffective against U23674. Therapy assignment via exome sequencing alone would thus have limited clinical utility for U23674.

#### Probabilistic target inhibition map (PTIM) modeling identifies 2-drug combinations with synergy in vitro

**Fig. 2:**
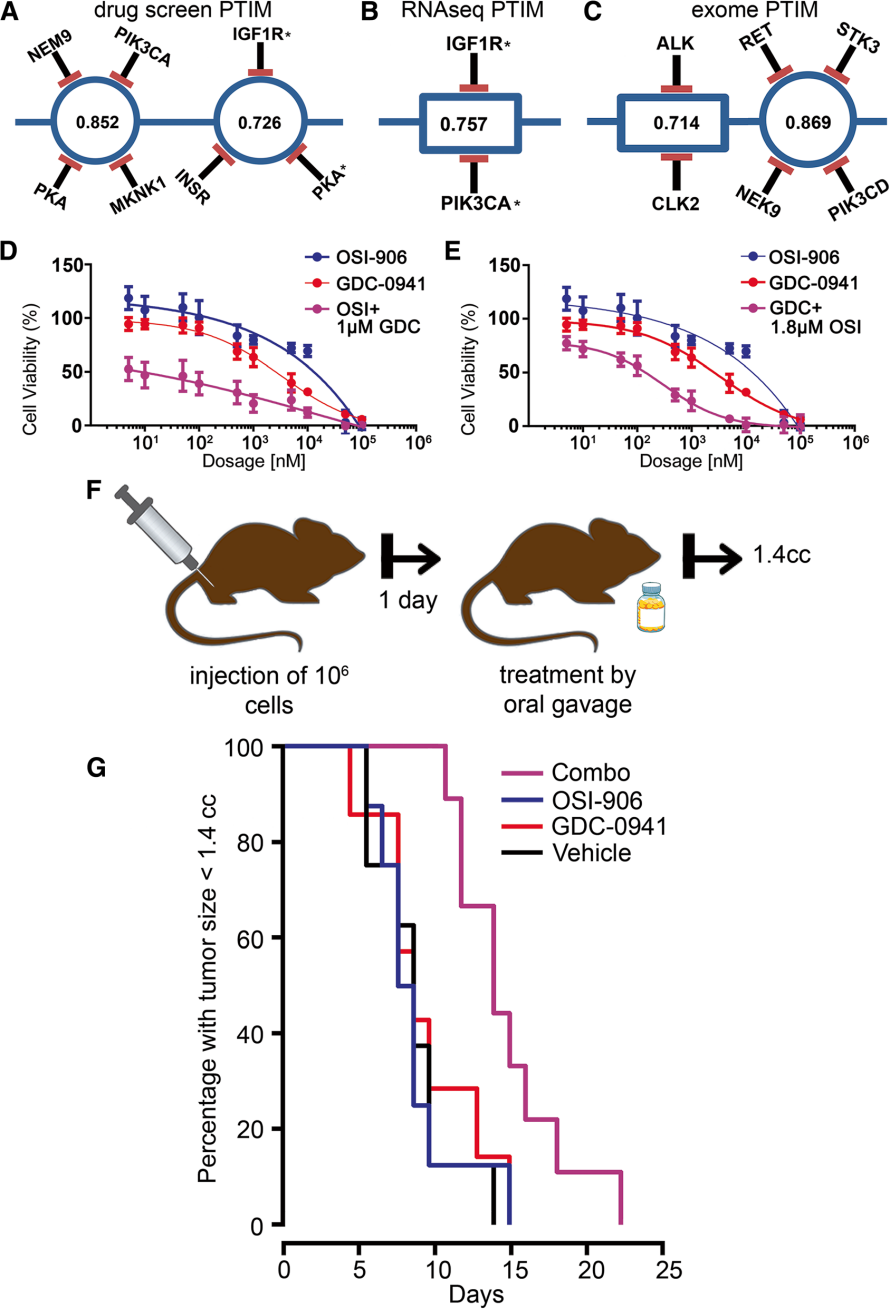
Probabilistic Target Inhibition Maps (PTIMs) and experimental in vitro and in vivo results for U23674 alveolar rhabdomyosarcoma (aRMS) drug combinations**.** Targets with adjacent asterisks indicate targets selected for in vitro validation. Values in the center of PTIM blocks represent expected scaled sensitivity following inhibition of associated block targets. **a** Abbreviated baseline chemical screen-informed PTIM. **b** Abbreviated chemical screen RNA-seq + informed PTIM. **c** Abbreviated chemical screen + exome-seq informed PTIM. The values within the target blocks indicate scaled drug sensitivity for the given target combination [16] when the targets are inhibited via one or more chemical compounds. More information can be found in prior publications [16, 18]. In (**d-e**), results are based on *n* = 3 technical replicates with *n* = 4 replicates per treatment condition. **d** Dose response curve for OSI-906 varied dosage + GDC-0941 fixed dosage. The response for GDC-0941 at varied dosages is included. **e** Dose response curve for GDC-0941 varied dosage + OSI-906 fixed dosage. The response for OSI-906 at varied dosages is included. **f** Schematic representation of in vivo experiment design. **g** Kaplan-Meier survival curves for in vivo orthotropic mouse experiment. Mice were treated with vehicle (*n* = 8 mice, black line), 50 mg/kg OSI-906 (*n* = 8 mice, blue line), 150 mg/kg GDC-0941 (*n* = 7 mice, red line), or combination 50 mg/kg OSI-906 + 150 mg/kg GDC-0941 (*n* = 8 mice, purple line). The medicine bottle image is public domain, provided by user Kim via clker.com (http://www.clker.com/clipart-blank-pill-bottle-3.html)

The high average level of target coverage (24 compounds/target), the inclusion of both typical and atypical kinase target combinations, and the thorough characterization of drug-target interactions made the GSK screen the most complete dataset available and was thus selected to guide in vitro and in vivo validation experiments. Baseline (chemical screen data only), RNA-seq informed-, exome-seq informed, siRNA interference informed-, and phosphoproteomics informed-PTIM models were generated from the GSK screen data (Fig. 2a-c**,** Additional file 5: Figure S5, Additional files 24, 25, 26, 27: Tables S10–S13). PTIM-identified targets were consistent with known targets of interest in aRMS [40, 41] and identified gene targets involved in established protein-protein interactions [42] (Additional file 6: Figure S6). As multi-drug combinations impart toxicity concerns and dosing limitations, we focus on PTIM blocks (combinations of two or more targets) treatable by at most two drugs. Baseline and genomics-informed PTIM models were also generated for the PPTI and Roche screens (Additional file 7: Figure S7, Additional file 27: Table S13), however no validation experiments based on PPTI or Roche PTIM models were performed due to focus on the GSK screen results.

We selected two combinations for in vitro synergy validation: 1) the RNA-seq-informed target combination *Igf1r* & *Pik3ca* (Fig. 2b) with combination therapy OSI-906 + GDC-0941 (a Pik3ca inhibitor selective against Akt/mTOR), and 2) The baseline target combination *Igf1r* & *Insr* & *Pka* with combination therapy OSI-906 (an Igf1r and Insr inhibitor) + SB-772077-B (Pka inhibitor, denoted GSK-PKA in figures). All compounds were selected based solely on selectivity of interaction with the PTIM-identified targets.

We selected the RNA-seq-informed drug combination due to high block sensitivity, targetability by a two-drug combination, and our previous work showing higher correlation between transcriptome status and drug sensitivity [14]. The baseline combination was selected due to targetability by a two-drug combination, higher score compared to other two-drug options, and to serve as a comparison between baseline PTIM models and molecularly-informed models. In vitro validation experiments for OSI-906 + GDC-0941 (Fig. 2d-e) demonstrated synergy as determined by non-constant ratio Combination Index [43] (CI) values (Additional file 28: Table S14). Low-dose combination experiments were also performed to confirm PTIM-predicted drug mechanism of action (Additional file 8: Figure S8, Additional file 28: Table S14). Both full-dose and low-dose OSI-906 + SB-772077-B in vitro validation experiments (Additional file 9: Figure S9) demonstrated non-constant ratio Combination Index synergy (Additional file 28: Table S14), though overall cell viability of was OSI-906 + SB-772077-B than higher the RNA-seq-informed combination. In vitro results support the potential of baseline and molecularly-informed PTIM modeling to discover synergistic target combinations, though inclusion of molecular data may narrow focus on targets which are overexpressed and/or aberrant and thus more likely to respond to drug treatment.

#### Tumor cell rewiring following synergy-focused combination therapy

To explore tumor rewiring (activation of secondary signaling pathways to improve chance of survival) following synergy-focused intervention, we treated U23674 cell populations with low-dose monotherapy or combination therapies defined in initial in vitro validation experiments, and subsequently screened the populations via the Roche screen (Additional files 10 and 11: Figures S10 and S11 and Additional file 29: Table S15). Unsurprisingly, the cell populations showed evidence of rewiring within hours of monotherapy or combination therapy intervention (Additional file 12: Figure S12, Additional files 27 and 28: Tables S13 and S14), emphasizing the importance of simultaneous, multi-pathway drug combinations at full therapeutic doses. While PTIM modeling currently focuses on 2-drug combinations to minimize toxicity concerns, PTIM-predicted combinations of three or more drugs are possible with sufficient evidence of safety and efficacy.

#### Probabilistic target inhibition map (PTIM) modeling predicts 2-drug combination with in vivo efficacy

Having demonstrated in vitro synergy, we next validated OSI-906 + GDC-0941 in vivo. We designed a four-arm orthotopic allograft study (Fig. 2f) comparing vehicle, OSI-906 (50 mg/kg), GDC-0941 (150 mg/kg), and OSI-906 (50 mg/kg) + GDC-0941 (150 mg/kg). Kaplan-Meier survival analysis (Fig. 2g) showed improvement in mouse lifespan from combination treatment (under Bonferroni correction: Vehicle – Combo, *p* = 0.005, OSI-906 – Combo, *p* = 0.014, GDC-0941 – Combo, *p* = 0.079. In all cases, *p* < 0.05 uncorrected). Survival of mice treated with either OSI-906 or GDC-0941 alone was indistinguishable from treatment by vehicle (*p* > 0.5, both corrected and uncorrected). Since a PTIM block represents targets which are weak independently but synergistic together, U23674 in vivo data supports the hypothesis underlying our modeling approach: synergistic combination targets can be identified through computational modeling of monotherapy chemical agents.

### Proof-of-concept of heterogeneity-consensus 2-drug combinations predicted by PTIM modeling

#### Development of heterogeneous cell models of Epithelioid Sarcoma (EPS)

EPS is a soft tissue sarcoma of children and adults for which chemotherapy and radiation provides little improvement in survival [44]. Effective options beyond wide surgical excision are presently undefined [45], making EPS a viable test case for developing targeted personalized therapies.

**Fig. 3:**
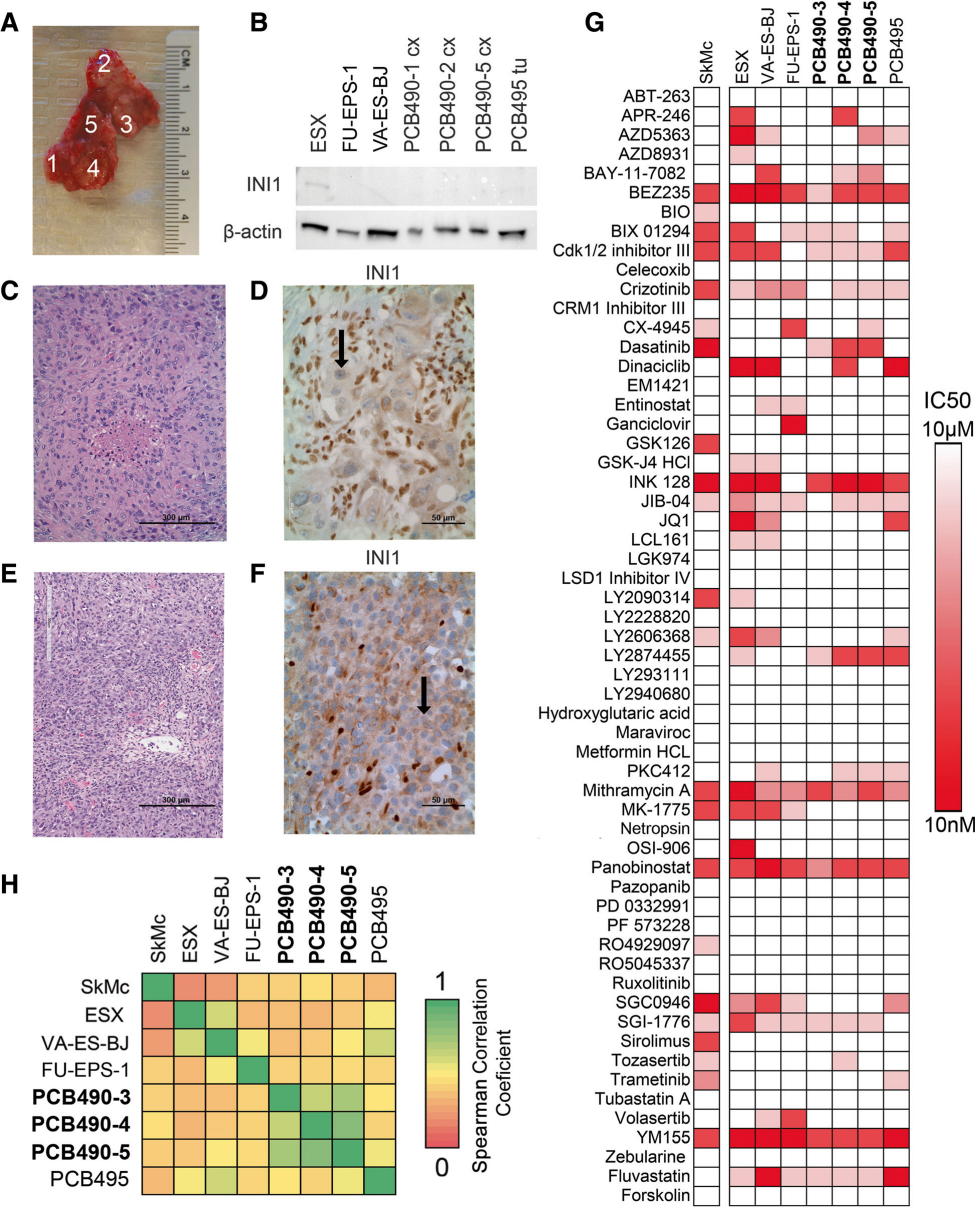
New cell cultures and patient-derived xenograft model of EPS with chemical space characterization**. a** PCB490 biopsy sample divided into distinct regions to create different primary tumor cell cultures for study. **b** Western blot demonstrating loss of INI1 in multiple primary tumor sites and in published EPS cell lines. **c** Histology of surgical biopsy of PCB490. **d** Immunohistochemical staining of PCB490 for INI1 shows absence in tumor cells (black arrow) but presence in co-mingled non-cancerous cells. **e** Histology of PCB490 patient-derived xenograft. **f** INI1 absence (black arrow) in immunohistochemical staining of PCB490 patient-derived xenograft. **g** Drug Screen V3 results from primary EPS cell cultures, published EPS cell lines, and a normal myoblast cell line. The heat values indicate drug sensitivity as IC50 values, scaled between 10 nM (red) and 10 μM (white, representing no IC_50_ achieved) **h** Heatmap of Pearson correlation coefficients of 60-agent drug screen results between a normal myoblast cell line (SkMC), three EPS cell lines (ESX, VA-ES-BJ, FU-EPS-1), three sites from PCB490 (PCB490–3, PCB490–4, PCB490–5), and an additional EPS patient-derived culture (PCB495). The heat values correspond to Pearson correlation coefficients between drug sensitivities of different cell models

We have developed several new heterogeneous EPS preclinical resources: three new unpublished cell cultures, as well as (to our knowledge) the first reported patient-derived xenograft (PDX) model of EPS derived from a 22-year-old female with a large proximal (shoulder) EPS tumor (Fig. 3a). The tumor sample was obtained from surgical resection and was assigned the internal identifier PCB490. Due to the size of the acquired tumor sample and the potential for heterogeneity in solid tumors [46], we divided the ~3 cm^2^ resected tumor mass into five spatially-distinct regions (designated PCB490–1 through PCB490–5) and cultured each region to develop heterogeneous cell models (Fig. 3a). PCB490 cultures were maintained at low passage to minimize biological drift from the original patient samples. To confirm EPS diagnosis, three of five tumor sites (1, 2, and 5) were validated by western blot for INI1 protein, which is absent in 93% of EPS samples (Fig. 3b) [44] as well as in published cell lines [21, 22]. Multiple sites were submitted to The Jackson Laboratory for establishment of PDX models; PCB490–5 PDX developed a passageable tumor that matched the original PCB490–5 sample by both histology and INI1 immunohistochemical staining (Fig. 3c-f).

#### Drug screening, sequencing, and comparison of heterogeneous EPS cell cultures

Cell cultures PCB490–3, PCB490–4, and PCB490–5 grew to sufficient populations (minimum 3 × 10^6^ cells) at low passage (passage 2 or below) to allow for drug screening via the investigator-selected 60-agent screen denoted Drug Screen V3 (Fig. 3g, Additional file 30: Table S16) and the previously described Roche screen (Additional file 31: Table S17). Drug screen endpoints were per-drug IC_50_ values.

PCB490 primary tissue was sequenced for tumor whole exome sequencing, matched normal whole exome sequencing, and whole transcriptome sequencing (Additional file 13: Figure S13, Additional files 32 and 33: Tables S18 and S19). Sequencing identified germline and tumor amplified, expressed, high-impact druggable variants in two genes (*ABL1*, *NOTCH1*) and expressed, medium impact variants in three additional genes (*MDM4*, *PAK4*, *MAP4K5*). All five variants were identified in both tumor and normal (germline) samples. The *ABL1* variant was previously identified in the 1000 Genomes Project [47]. The *ABL1*, *NOTCH1*, *MDM4* and *PAK4* variants were previously submitted to the dbSNP database [48]. All variants are of unknown clinical significance (Additional file 34: Table S20) [48, 49]. PCB490 drug screening results revealed no pathway-specific drug sensitivity of mutated genes (Additional file 14: Figure S14) suggesting therapy assignment via exome sequencing alone would likely have limited clinical utility for PCB490.

To compare drug sensitivity of PCB490 with other EPS models, three cell lines (ESX, FU-EPS-1, and VA-ES-BJ), a second human-derived cell culture (PCB495), and the SkMc skeletal myoblast cell line were assayed with Drug Screen V3 (Fig. 3g, Additional file 35: Table S21). Drug Screen V3 responses were compared by calculating Spearman correlation coefficients (Fig. 3h) to quantify the similarity between the new EPS models and existing EPS cell models. For the purpose of this analysis, we treat the PCB490 cultures from different regions as independent samples. Correlation within primary cell cultures (PCB490 sites and PCB495) was significantly higher than correlation between primary cultures and cell lines (μ = 0.6466 vs. μ = 0.4708, *p* < 0.01), suggesting EPS primary cultures may be biologically distinct from EPS cell lines. PCB490 drug screen response differed between sample locations millimeters away from each other, reflective of biological differences arising from spatial tumor heterogeneity. Nonetheless, correlation between chemical screen results from PCB490 cultures was significantly higher than correlation between PCB490 cultures and PCB495 cultures/EPS cell lines (μ = 0.7671 vs. μ = 0.4601, p < 0.001), suggesting that treatments for PCB490 may be better defined solely by PCB490 biological data.

#### PTIM modeling guides heterogeneity-consensus in vivo drug combination

**Fig. 4:**
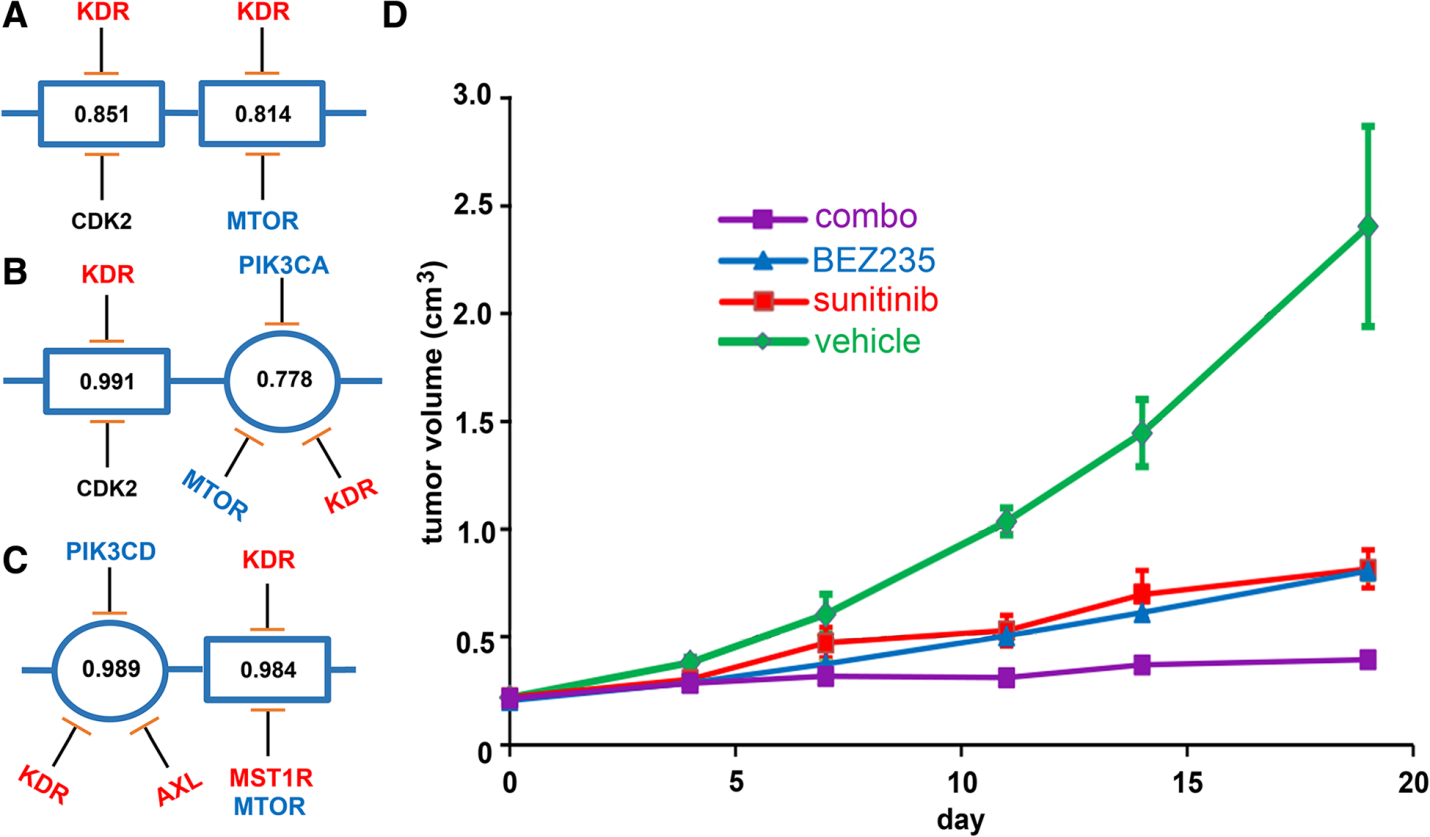
Probabilistic Target Inhibition Maps (PTIMs) of Drug Screen V3 and Roche screen results for spatially-distinct epithelioid sarcoma tumor regions**.** Values in the center of PTIM blocks represent expected scaled sensitivity following inhibition of associated block targets. **a-c** PTIMs informed by Roche Orphan Kinase Library and V3 screens. Targets of sunitinib are highlighted red, targets of BEZ235 are highlighted blue. **a** Abbreviated PTIM for PCB490–3. **b** Abbreviated PTIM for PCB490–4. **c** Abbreviated PTIM from PCB490–5 with integrated RNA-seq data. **d** Results from PCB490–5 patient-derived xenograft in vivo validation studies presented as group-wide tumor volumes following vehicle treatment (*n* = 3 mice, green line), treatment by 30.0 mg/kg sunitinib (*n* = 3 mice, red line), treatment by 25.0 mg/kg BEZ235 (*n* = 3 mice, blue line), and treatment by 25.0 mg/kg BEZ235 + 30.0 mg/kg sunitinib (*n* = 3 mice, purple line)

Highly correlated yet heterogeneous PCB490 drug sensitivity data guided us towards PTIM modeling to design a heterogeneity-consensus personalized drug combination therapy for PCB490. PTIM models of PCB490–3 (Fig. 4a, Additional file 27: Table S13), PCB490–4 (Fig. 4b, Additional file 27: Table S13), and PCB490–5 with integrated RNA-seq data (Fig. 4c, Additional file 27: Table S13) indicated common efficacious mechanisms across the heterogeneous tumor sites: epigenetic modifiers (HDAC, EHMT), PI3K/mTOR inhibition, and VEGF (KDR) signaling inhibition. We focused on high-scoring PTIM blocks treatable by a two-drug combination, resulting in selection of BEZ235 (PI3K/mTOR inhibitor) and sunitinib (poly-kinase inhibitor, including KDR and AXL). BEZ235 + sunitinib was selected solely based on PTIM modeling data, agnostic to previous use of sunitinib in EPS [50].

To replicate potential clinical conditions for personalized combination therapy, we bypassed in vitro validation and directly initiated in vivo testing of BEZ235 + sunitinib in the PCB490 PDX model. Though the PCB490 PDX model originates from the PCB490–5 region, heterogeneity of PCB490 suggests the tumor section used to establish the PCB490 PDX can be considered a unique heterogeneous region. PDX testing of BEZ235 + sunitinib demonstrated significant slowing of tumor growth over vehicle control (92% slower tumor growth at Day 19, *p* = 0.01) (Fig. 4d). In statistical analysis restricted to treated animals at Day 19, BEZ235 + sunitinib significantly slowed PDX tumor growth compared to both BEZ235 (p = 0.01) and sunitinib (p = 0.01) alone (Fig. 4d).

### Proof-of-concept of resistance-abrogating 2-drug combinations predicted by PTIM modeling

#### PTIM modeling of undifferentiated pleomorphic sarcoma (UPS) samples guides cross-species resistance-abrogating drug combination in vitro

**Fig. 5:**
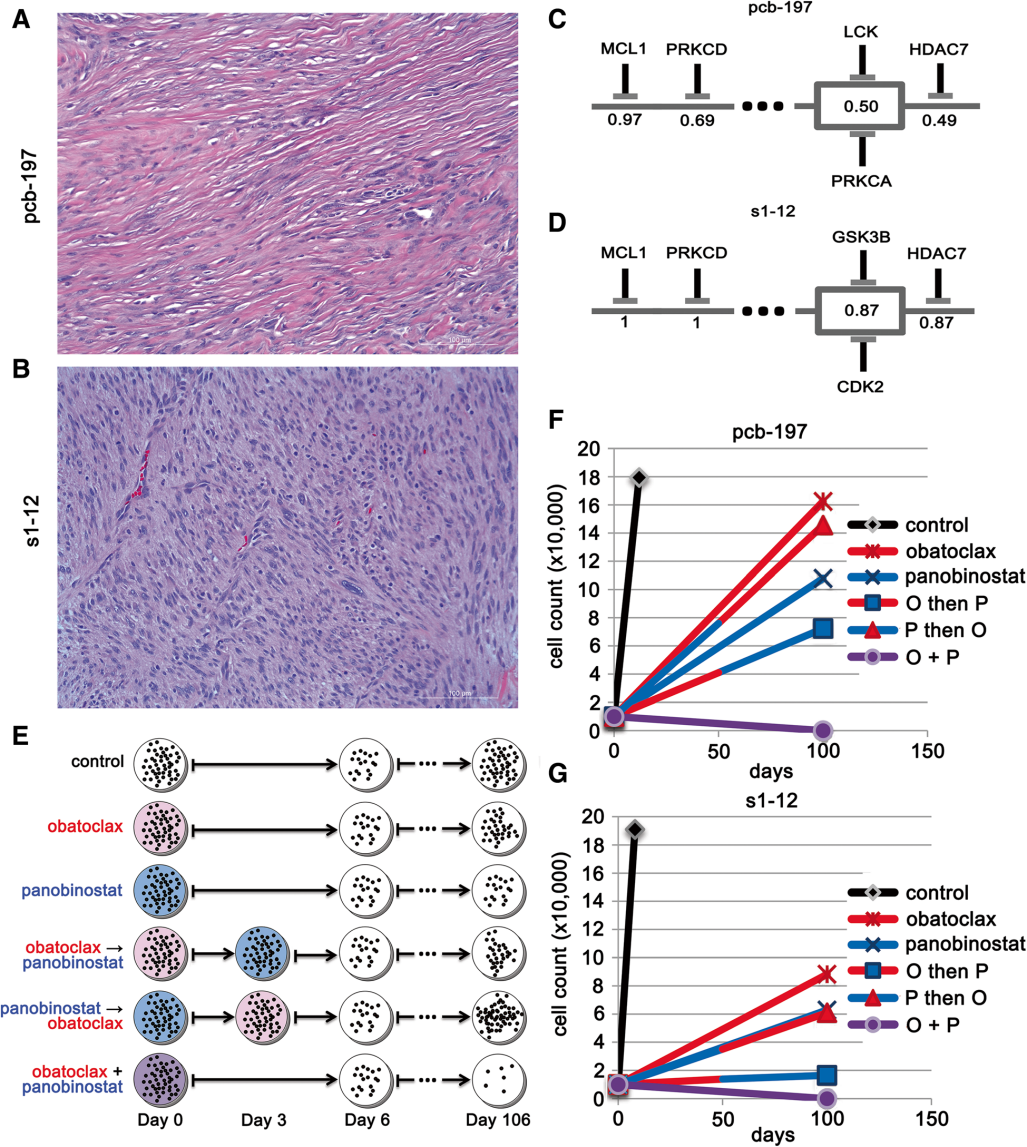
Undifferentiated pleomorphic sarcoma (UPS) Probabilistic Target Inhibition Map (PTIM)-guided resistance abrogation experiments. Values in the center of PTIM blocks represent expected scaled sensitivity following inhibition of associated block targets. **a** Histology of PCB197 human UPS sample (20x magnification). **b** Histology of S1–12 canine UPS sample (20x magnification). **c** Abbreviated PTIM model for the pediatric preclinical testing initiative (PPTI) screen of PCB197 human UPS sample. **d** Abbreviated PTIM model built from the PPTI screen of S1–12 canine UPS sample. **e** Schematic of experimental design for resistance abrogation experiments. **f** Cellular regrowth of PCB197 human UPS sample over 100 days following treatment by single and multi-agent compounds in sequence and in combination. **g** Cellular regrowth of S1–12 canine UPS sample over 100 days following treatment by single and multi-agent compounds in sequence and in combination. Data in (**f-g**) is based on *n* = 4 replicate experiments

The previously discussed U23674 rewiring experiment emphasized the need for multi-pathway targeting when developing personalized treatments. The PTIM modeling approach identifies mechanisms driving in vitro drug sensitivity by identifying effective target combination “blocks”; two blocks operating on different biological pathways represent two independent treatment mechanisms. We reasoned that two-block inhibition could result in resistance-abrogating combination treatments, thus we validate a drug combination designed from two PTIM blocks representing independent biological pathways. PTIM modeling of PPTI screen data from a UPS derived from a 75-year-old man (PCB197, Fig. 5a, Additional file 36: Tables S22) and a canine-origin UPS (S1–12, Fig. 5b, Additional file 36: Table S22) identified species-consensus drug sensitivity mechanisms targetable by a 2-block, 2-drug combination (Fig. 5c, d, Additional file 27: Table S13): panobinostat (pan-HDAC inhibitor, HDAC7 block) and obatoclax (MCL1 inhibitor). The combination of panobinostat + obatoclax was predicted to abrogate resistance mechanisms and prevent cancer cell rewiring and regrowth; furthermore, the cross-species nature of the experiment supports the resistance-abrogation effect not being model specific.

To validate in vitro resistance abrogation across species, we performed identical six-arm in vitro trials for PCB197 and S1–12. Each arm represented a different combination method for the cross-species combination: vehicle treatment, monotherapy treatment, serial monotherapy treatment (panobinostat then obatoclax, obatoclax then panobinostat), and simultaneous combination treatment (concurrent panobinostat + obatoclax) (Fig. 5e). Resistance abrogation in each arm was determined by cellular regrowth over 100 days following treatment. Rewiring and regrowth was expected for all monotherapy and serial treatment modalities. All arms except the simultaneous combination treatment arm experienced cellular regrowth, indicating the development of resistance. In both cultures, the simultaneous combination treatment arm showed no cellular regrowth over 100 days, indicating the combination potentially addressed resistance mechanisms (Fig. 5f, g).

## Discussion

The work presented here represents validation experiments for three aspects of PTIM-guided personalized cancer therapy design: drug sensitivity and synergy prediction in a GEMM-origin aRMS, heterogeneity-consensus drug combination design and validation in the first-reported EPS PDX model, and mitigation of cancer cell resistance mechanisms in cross-species in vitro validation experiments. Our studies suggest the high value of combining functional screening data with secondary molecular data (especially RNA-seq data) in the design of personalized drug combinations as a supplement to or alternative to DNA sequencing-based therapy assignment. While increased effort is required to generate functional data, the additional information and evidence may prove useful in designing therapeutically effective personalized cancer treatments.

Critically, the timeframe for PTIM-based combination therapy design is less than the time required for standard high-throughput sequencing experiments. The PTIM analysis pipeline can be performed in under 2 weeks and without the explicit need for sequencing results. Currently, the time limiting step in integrative PTIM analysis is exome and RNA sequencing, for which new technology is rapidly reducing time and monetary cost. Functional drug screening in standard well plates can be performed for under $300, and CLIA-certified physical sequencing experiments are now under $500 per analyte per experiment; the cost of a complete functional and molecular analysis now represents a fraction of drug cost and may be accessible to a large population of cancer patients.

The three PTIM-guided validation experiments serve as proofs-of-concept rather than a full clinical validation. The current study lacks the large sample size necessary to reach definite conclusions on the large-scale efficacy of PTIM-based personalized cancer therapy. Any treatment strategy, especially a personalized approach, requires a large population to draw clinically-relevant conclusions. Increasing the sample size of personalized treatments designed by the PTIM approach is required to demonstrate clinical use. To that end, the critical next stage in PTIM-based personalized therapy design will be prospective evaluation by partnering with physicians and veterinarians to pilot testing of n-of-1 personalized therapies in individual human patients and animals with spontaneous cancer. As the cost of analysis is low, the major challenges will be 1) administration of FDA-approved drugs, very likely as off-label therapy in combinations potentially not validated in Phase I trials, and 2) financial costs associated with modern targeted therapy regimens, which may currently be prohibitive for some patients.

As drug screen results ultimately guide PTIM modeling, computational modeling of different disease types will require designing disease-specific compound screens to maximize the breadth and depth of disease-relevant multi-target interactions [15]. Similarly, different types of secondary molecular data influences target selection during PTIM model construction depending on the underlying analyte or perturbation, with different secondary datasets expectedly producing different PTIM models. Selection of secondary datasets to generate for individual cases will depend on availability of tumor tissue and expected predictive utility of individual datasets. Based on widespread clinical utility and published studies, the current standard secondary datasets for PTIM modeling are exome sequencing data and RNA sequencing data [14]. As high-throughput analysis of additional biological analytes becomes available through CLIA certified procedures, new datatypes will be integrated into PTIM models. In particular, recent advances in robust generation of proteomics data from patient samples [51–53] may enable routine integration of proteomics data into PTIM modeling beyond the test case presented in this work.

PTIM-based personalized cancer therapy also requires development of personalized toxicity and dosing prediction methods for designing maximally effective, minimally toxic drug combinations. Research on toxicity prediction is underway, as is research on incorporating chemotherapy backbone into drug combination predictions. While validated PTIM models are currently based on low-passage cell cultures (U23674, PCB490, S1–12, PCB197), future application of PTIM models will use direct-to-plate tumor screening to best recapitulate the patient’s disease state and to remove the dependence on cell culture establishment. Finally, we will pursue expansion of disease-consensus PTIM modeling [19] to establish new disease-specific drug combinations based on integrated drug screening and high-throughput sequencing data.

## Conclusion

PTIM-based personalized combination therapy has been designed to uniquely leverage patient-specific functional and biological data to address some of the critical unmet clinical needs of the 60% of cancer patients for whom tumor DNA analysis is uninformative [2] and the 600,000 patients lost to cancer every year [1] who have exhausted clinical options. PTIM modeling can also meet the needs of cancer patients with rare diseases, such as the spectrum of 60+ cancers known as non-rhabdomyosarcoma soft tissue sarcomas (including EPS) for which effective clinical treatments may not exist and may never be developed due to a paucity of disease models for research. These two groups represent a significant cancer patient population for which PTIM modeling may provide evidence-based treatment options where no therapeutic avenues exist.

## Additional files

Additional file 1: **Figure S1.** Heat map of merged chemical screen, RNA-seq, siRNA, and phosphoproteomics results for GlaxoSmithKline (GSK) Orphan Kinome screen. Due to the large number of compounds and protein targets, only a limited scope of compounds and targets is shown here (for full data, see Additional file 15: Table S1). Bright red indicates high sensitivity values, gradating down to white meaning low sensitivity. Gray indicates no interaction or no available data. Asterisk indicates targets later validated in vivo. (TIF 38030 kb)

Additional file 2: **Figure S2.** Heat map of merged Roche Orphan Kinome chemical screen, RNA-seq, siRNA, and phosphoproteomics results. Due to the large number of compounds and protein targets, only a limited scope of compounds and targets is shown here (For full data, see Additional file 17: Table S3). Bright red indicates high sensitivity values, gradating down to white meaning low sensitivity. Gray indicates no interaction or no available data. (TIF 58505 kb)

Additional file 3: **Figure S3.** Heat map of joint version 2.1 chemical screen, RNA-seq, siRNA, and phosphoproteomics results. Due to the large number of compounds and protein targets, only a limited scope of compounds and targets is shown here (For full data, see Additional file 21: Table S7). Bright red indicates high sensitivity values, gradating down to white meaning low sensitivity. Gray indicates no interaction or no available data (TIF 57387 kb)

Additional file 4: **Figure S4.** Circos plot of U23674 RNA sequencing and exome sequencing data. The outermost data circle represents log_2_-scaled gene expression [log_2_(expression+ 1), low expression (white) to high expression (red), with missing values colored black]. The middle circle represents genes with identified mutations or indels (red) or lack thereof (black). The innermost circle represents copy number variations (red is amplification, blue is deletion, black is no variation). (TIF 67243 kb)

Additional file 5: **Figure S5.** PTIM models developed using secondary datasets. (**A**) Chemical screen + siRNA informed PTIM. Values in the center of PTIM blocks represent expected scaled sensitivity following inhibition of associated block targets. (**B**) Chemical screen + phosphoproteomics informed PTIM. The values within the target blocks indicate scaled drug sensitivity [16] when block targets are inhibited. (TIF 22272 kb)

Additional file 6: **Figure S6.** STRINGdb visualizations of protein-protein interaction networks implicated by PTIM models. The protein-protein interaction networks here are derived from targets selected to define drug sensitivity during PTIM modeling. Edges in the STRINGdb graph represent confidence of interactions based on data from multiple published sources. Edges with confidence > 0.9 are represented on the graph. The asterisk indicates targets validated in vitro*.* (**A**) Network of the set of targets common to the models developed for the GSK Orphan Kinome screen and the PPTI screen. Enrichment *p*-value < 0.01. (**B**) Network of the targets identified by the GSK Orphan Kinome screen alone. Enricment p-value < 0.01. (TIF 27210 kb)

Additional file 7: **Figure S7.** Probabilistic Target Inhibition Map (PTIM) model of U23674 Roche chemical screen hits. Values in the center of PTIM blocks represent expected scaled sensitivity following inhibition of associated block targets. (A) Base chemical screen informed PTIM. (B) RNA-seq + chemical screen informed PTIM. Roche screen hits include CDK2 inhibitors. However, no CDK inhibitor was a known inhibitor of non-CDK targets, limiting development of personalized combinations involving CDK inhibitors. (TIF 29510 kb)

Additional file 8: **Figure S8.** Low dose combination validation results for drug combinations GDC-0941 + OSI-906. Results are based on *n* = 3 technical replicates with *n* = 4 replicates per treatment condition. (**A**) Dose response curve for OSI-906 varied dosage + GDC-0941 low fixed dosage. The response for GDC-0941 at varied dosages is included. (**B**) Dose response curve for GDC-0941 varied dosage + OSI-906 low fixed dosage. The response for OSI-906 at varied dosages is included. (TIF 10578 kb)

Additional file 9: **Figure S9.** Combination validation results for drug combinations SB-772077-B (GSK-PKA) + OSI-906. Results are based on *n* = 3 technical replicates with *n* = 4 replicates per treatment condition. (**A**) Dose response curve for OSI-906 varied dosage + SB-772077-B fixed dosage. Response for SB-772077-B at varied dosages is included. (**B**) Dose response curve for OSI-906 varied dosage + SB-772077-B low fixed dosage. (**C**) Dose response curve for SB-772077-B varied dosage + OSI-906 fixed dosage. The response for OSI-906 at varied dosages is included. (**D**) Dose response curve for SB-772077-B varied dosage + OSI-906 low fixed dosage. The response for OSI-906 at varied dosages is included. (TIF 67346 kb)

Additional file 10: **Figure S10.** Schematic of PTIM-informed U23674 rewiring experiment. An initial culture of U23674 is screened using the Roche screen. The same culture is used to seed 6 new cultures, which are grown until the cell population is sufficient for drug screening. Five of the 6 cultures were treated using single agents and drug combinations in low dosages (75 nM OSI-906, 50 nM GDC-0941) and one culture was left untreated. After treatment and incubation for 72 h, the compounds were removed the cells were screened using the Roche Orphan Kinome screen. (TIF 8496 kb)

Additional file 11: **Figure S11.** Heat map of joint Roche Orphan Kinome chemical screen, RNA-seq, siRNA, and phosphoproteomics results from the U23674 Probabilistic Target Inhibition Map (PTIM) rewiring experiment. Due to the large number of compounds and protein targets, only a limited scope of compounds and targets is shown here (For full data, see Additional file 28: Table S14). Bright red indicates high sensitivity values, gradating down to white meaning low sensitivity. Gray indicates no interaction or no available data. (TIF 96004 kb)

Additional file 12: **Figure S12.** Probabilistic Target Inhibition Map (PTIM) models from U23674 experimental rewiring data. Values in the center of PTIM blocks represent expected scaled sensitivity following inhibition of associated block targets. (**A**) Untreated initial culture PTIM. (**B**) Untreated secondaryculture PTIM. (**C**) OSI-906-treated rewire PTIM. (**D**) GDC-0941-treated rewire PTIM. (**E**) OSI-906 + GDC-0941-treated rewire PTIM. (TIF 41768 kb)

Additional file 13: **Figure S13.** Circos plot of PCB490 RNA sequencing and exome sequencing data. *ABL1* and *NOTCH1* are identified as both mutated and amplified, though both variants were also identified in the matched germline sample. The outermost data circle represents log_2_-scaled gene expression [log_2_(expression+ 1), low expression (white) to high expression (red), with missing values colored black]. The middle circle represents genes with identified mutations or indels (red) or lack thereof (black). The innermost circle represents copy number variations (red is amplification, blue is deletion, black is no variation). (TIF 69008 kb)

Additional file 14: **Figure S14.** Heat map of IC_50_ and EC_50_ values for Pediatric Preclinical Testing Initiative Version 3 drug screen compounds inhibiting mutated and expressed targets in PCB490. Red in the IC_50_ and EC_50_ tables indicates low IC_50_ and EC_50_ values, respectively. No single target or combination of targets showed uniform efficacy across all PCB490 cultures, suggesting variations alone or in conjunction with transcriptome sequencing would not have identified actionable therapeutic targets. Heat values in the IC_50_ section of the table represent drug sensitivities as IC_50_ values, between 1 nM (red) and 6 μM or above (white). Heat values in the EC_50_ section of the table represent quantified drug-target interaction between chemical agents and gene targets, quantified as 50% inhibitory concentrations between 1 nM (red) and 6 μM or above (white), with grey representing no interaction. (TIF 13895 kb)

Additional file 15: **Table S1.** Merged GSK Screen Data - U23674. (XLSX 271 kb)

Additional file 16: **Table S2.** GSK Screen Data IC50 Data - U23674. (XLSX 15 kb)

Additional file 17: **Table S3.** Roche Screen Merged Data - U23674. (XLSX 88 kb)

Additional file 18: **Table S4.** Roche screen hit references. (XLSX 13 kb)

Additional file 19: **Table S5.** Roche screen IC50 data - U23674. (XLSX 9 kb)

Additional file 20: **Table S6.** PPTI screen IC50 data - U23674. (XLSX 10 kb)

Additional file 21: **Table S7.** PPTI Screen Merged data - U23674. (XLSX 130 kb)

Additional file 22: **Table S8.** Exome Sequencing Data - U23674. (XLSX 1792 kb)

Additional file 23: **Table S9.** RNA Sequencing Data - U23674. (XLSX 2157 kb)

Additional file 24: **Table S10.** RAPID siRNA Screen data - U23674. (XLSX 43 kb)

Additional file 25: **Table S11.** Rapid Screen vs Drug screen - U23674. (XLSX 10 kb)

Additional file 26: **Table S12.** Phospho Screen data - U23674. (XLSX 657 kb)

Additional file 27: **Table S13.** PTIM Map Scores. (XLSX 14 kb)

Additional file 28: **Table S14.** Combination Index Values - U23674. (XLSX 13 kb)

Additional file 29: **Table S15.** Rewiring Screening Data - U23674. (XLSX 270 kb)

Additional file 30: **Table S16.** V3 Drug Screen data - PCB490. (XLSX 10 kb)

Additional file 31: **Table S17.** Roche Drug Screen data - PCB490. (XLSX 14 kb)

Additional file 32: **Table S18.** Exome Sequencing Data - PCB490. (XLSX 345 kb)

Additional file 33: **Table S19.** RNA Sequencing data - PCB490. (XLSX 3314 kb)

Additional file 34: **Table S20.** Druggable Exome Targets - PCB490. (XLSX 11 kb)

Additional file 35: **Table S21.** EPS Model V3 Drug Screen Data. (XLSX 11 kb)

Additional file 36: **Table S22.** PPTI Drug Screening Data – UPS. (XLSX 9 kb)

## Abbreviations

aRMS: Alveolar rhabdomyosarcoma
CCLE: Cancer Cell Line Encyclopedia
CCuRe-FAST: Childhood Cancer Registry for Familial and Sporadic Tumors
CI: Combination Index
DIPG: Diffuse intrinsic pontine glioma
EGA: European Genome-Phenome Archive
EPS: Epithelioid sarcoma
FBS: Fetal bovine serum
GEO: Gene Expression Omnibus
GSK: GlaxoSmithKline
IACUC: Institutional Animal Care and Use Committee
IHC: Immunohistochemistry
IRB: Institutional Review Board
JAX: The Jackson Laboratory
OHSU: Oregon Health & Science University
OSU: Oregon State University
PBS: Phosphate Buffered Saline
PDX: Dulbecco’s Modified Eagle’s Medium
PDX: Patient-derived xenograft
PPTI screen: Pediatric Preclinical Testing Initiative Drug Screen Version 2.1
PTIM: Probabilistic Target Inhibition Map
RIPA: Radioimmunoprecipitation
SD: Standard deviation
SHO: SCID/hairless/outbred mice
STR: Short Tandem Repeat
TV: Tumor volume
UPS: Undifferentiated pleomorphic sarcoma
