# Supplementary figures and images for: Probabilistic modeling of personalized drug combinations from integrated chemical screen and molecular data in sarcoma
Source: BMC Cancer. 2019 Jun 17;19:593. doi: 10.1186/s12885-019-5681-6 (PMC6580486; doi:10.1186/s12885-019-5681-6)

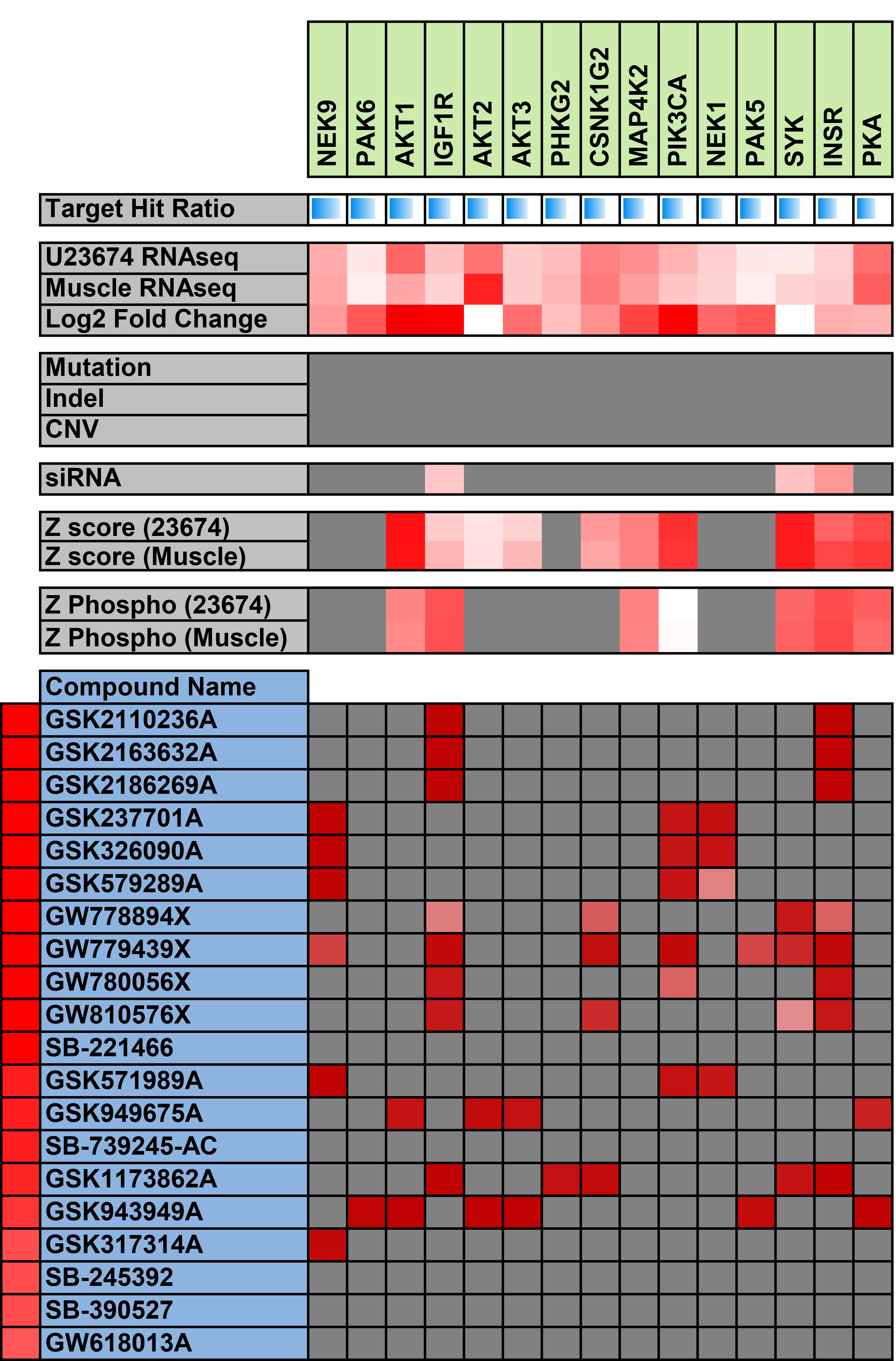

Supplement: Supplementary file 1 — Figure S1. Heat map of merged chemical screen, RNA-seq, siRNA, and phosphoproteomics results for GlaxoSmithKline (GSK) Orphan Kinome screen. Due to the large number of compounds and protein targets, only a limited scope of compounds and targets is shown here (for full data, see Additional file 15: Table S1). Bright red indicates high sensitivity values, gradating down to white meaning low sensitivity. Gray indicates no interaction or no available data. Asterisk indicates targets later validated in vivo. (TIF 38030 kb) [file 12885_2019_5681_MOESM1_ESM.tif]

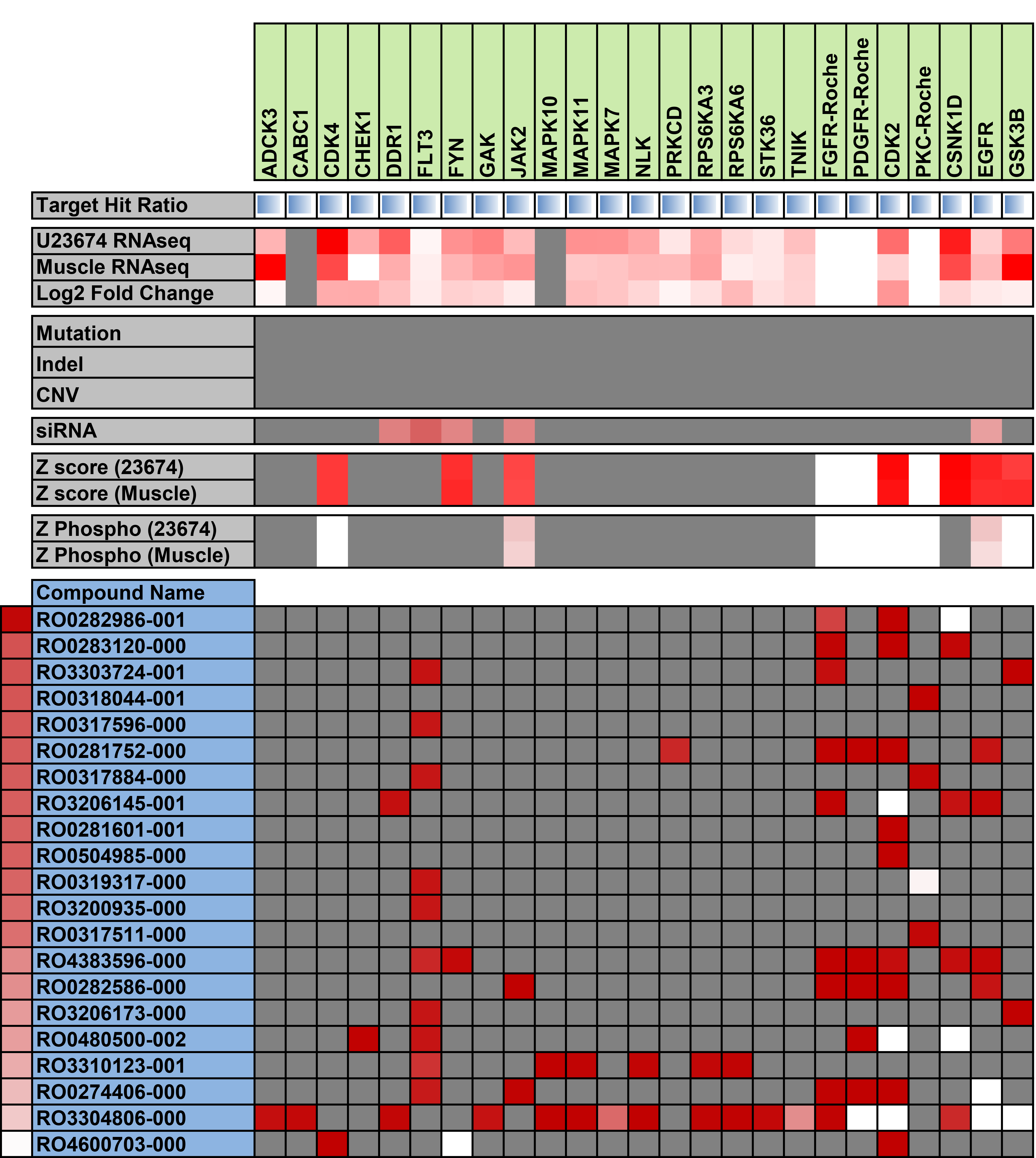

Supplement: Supplementary file 2 — Figure S2. Heat map of merged Roche Orphan Kinome chemical screen, RNA-seq, siRNA, and phosphoproteomics results. Due to the large number of compounds and protein targets, only a limited scope of compounds and targets is shown here (For full data, see Additional file 17: Table S3). Bright red indicates high sensitivity values, gradating down to white meaning low sensitivity. Gray indicates no interaction or no available data. (TIF 58505 kb) [file 12885_2019_5681_MOESM2_ESM.tif]

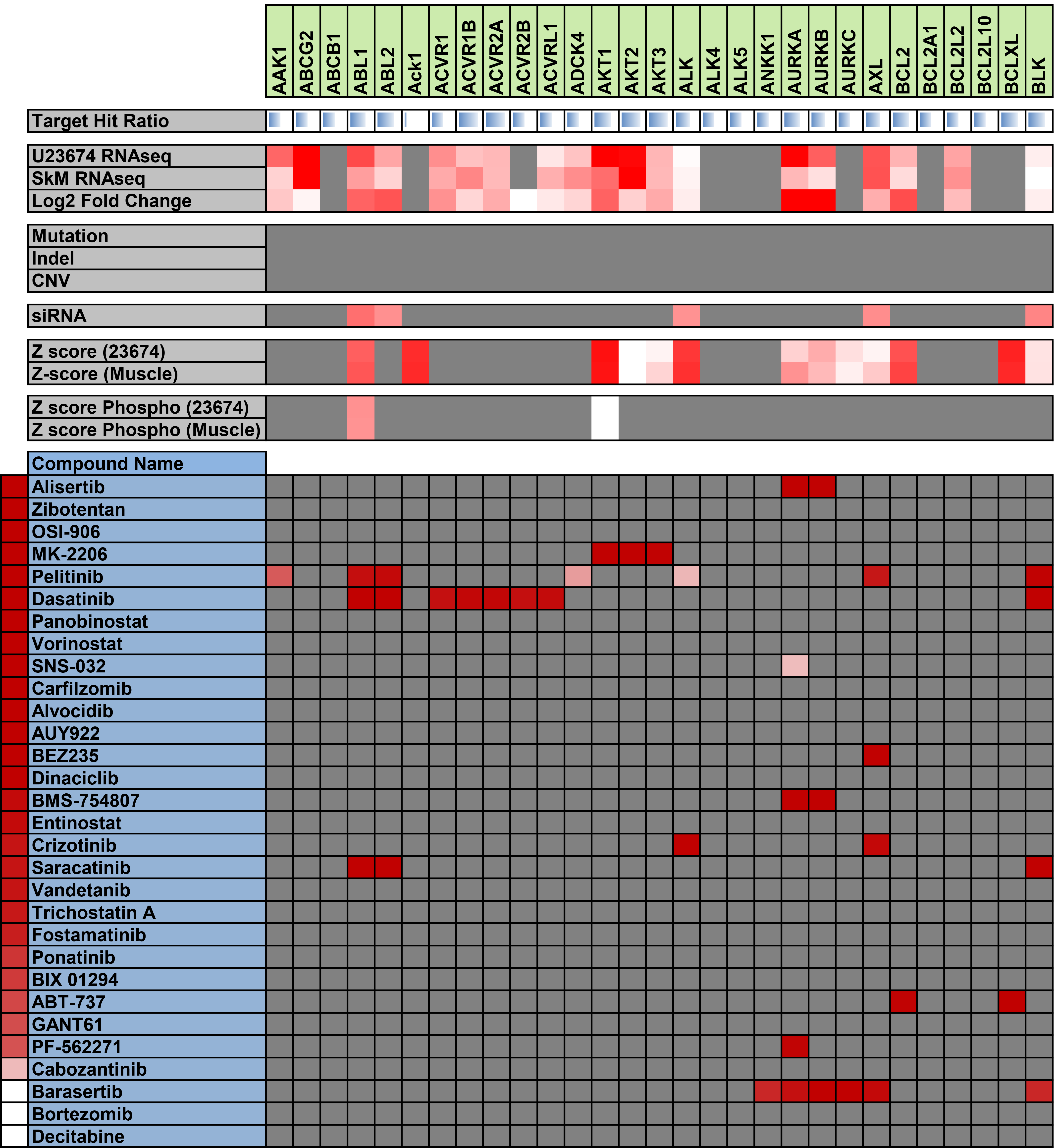

Supplement: Supplementary file 3 — Figure S3. Heat map of joint version 2.1 chemical screen, RNA-seq, siRNA, and phosphoproteomics results. Due to the large number of compounds and protein targets, only a limited scope of compounds and targets is shown here (For full data, see Additional file 21: Table S7). Bright red indicates high sensitivity values, gradating down to white meaning low sensitivity. Gray indicates no interaction or no available data (TIF 57387 kb) [file 12885_2019_5681_MOESM3_ESM.tif]

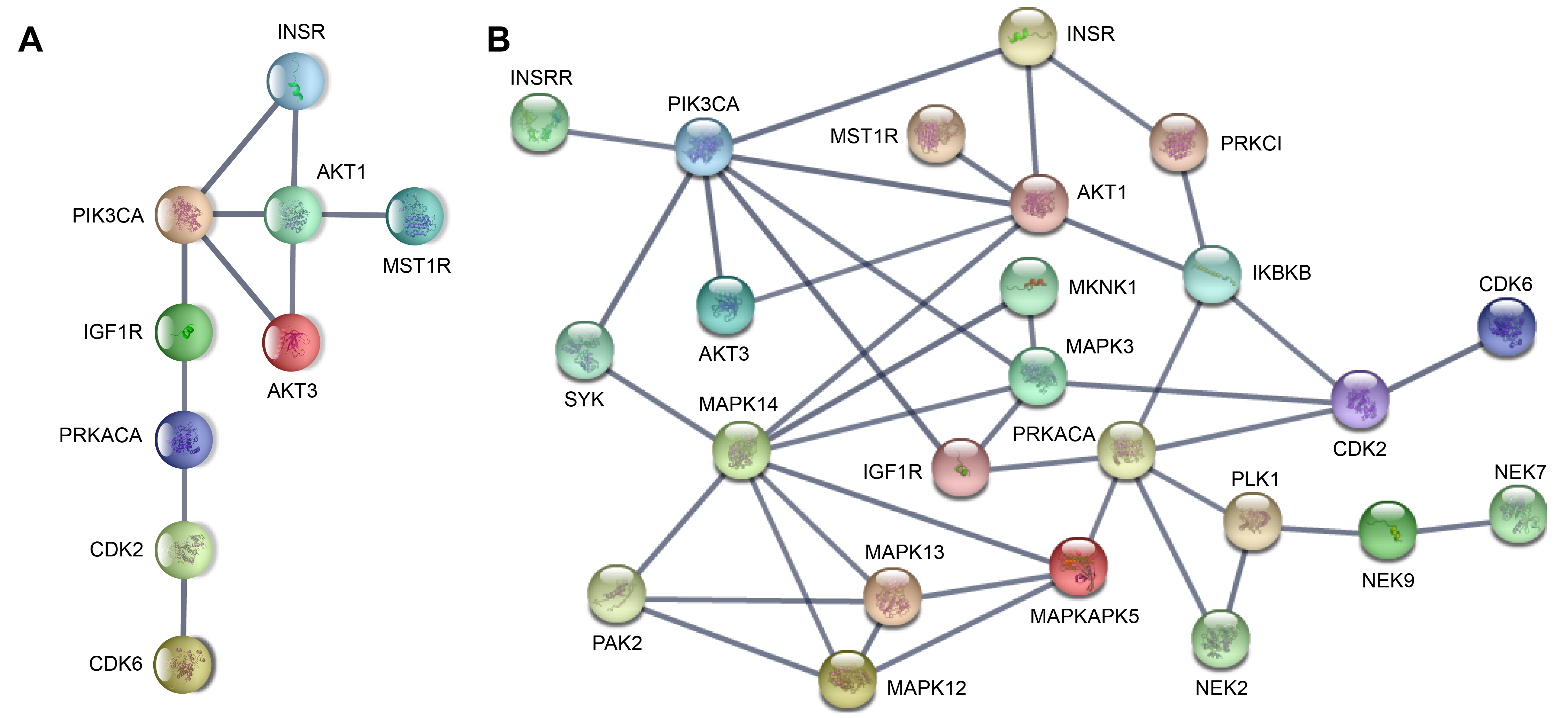

Supplement: Supplementary file 6 — Figure S6. STRINGdb visualizations of protein-protein interaction networks implicated by PTIM models. The protein-protein interaction networks here are derived from targets selected to define drug sensitivity during PTIM modeling. Edges in the STRINGdb graph represent confidence of interactions based on data from multiple published sources. Edges with confidence > 0.9 are represented on the graph. The asterisk indicates targets validated in vitro. (A) Network of the set of targets common to the models developed for the GSK Orphan Kinome screen and the PPTI screen. Enrichment p-value < 0.01. (B) Network of the targets identified by the GSK Orphan Kinome screen alone. Enricment p-value < 0.01. (TIF 27210 kb) [file 12885_2019_5681_MOESM6_ESM.tif]

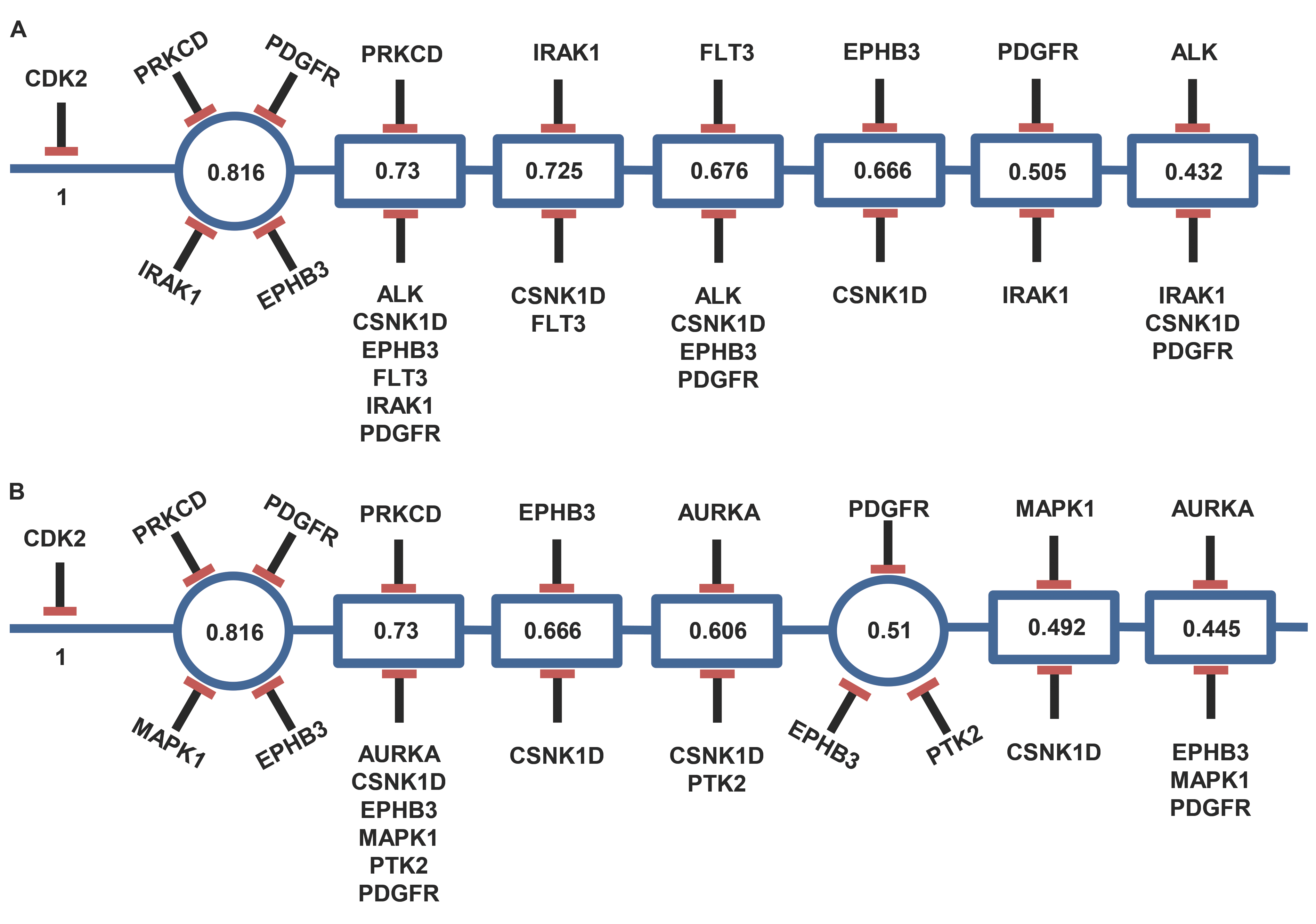

Supplement: Supplementary file 7 — Figure S7. Probabilistic Target Inhibition Map (PTIM) model of U23674 Roche chemical screen hits. Values in the center of PTIM blocks represent expected scaled sensitivity following inhibition of associated block targets. (A) Base chemical screen informed PTIM. (B) RNA-seq + chemical screen informed PTIM. Roche screen hits include CDK2 inhibitors. However, no CDK inhibitor was a known inhibitor of non-CDK targets, limiting development of personalized combinations involving CDK inhibitors. (TIF 29510 kb) [file 12885_2019_5681_MOESM7_ESM.tif]

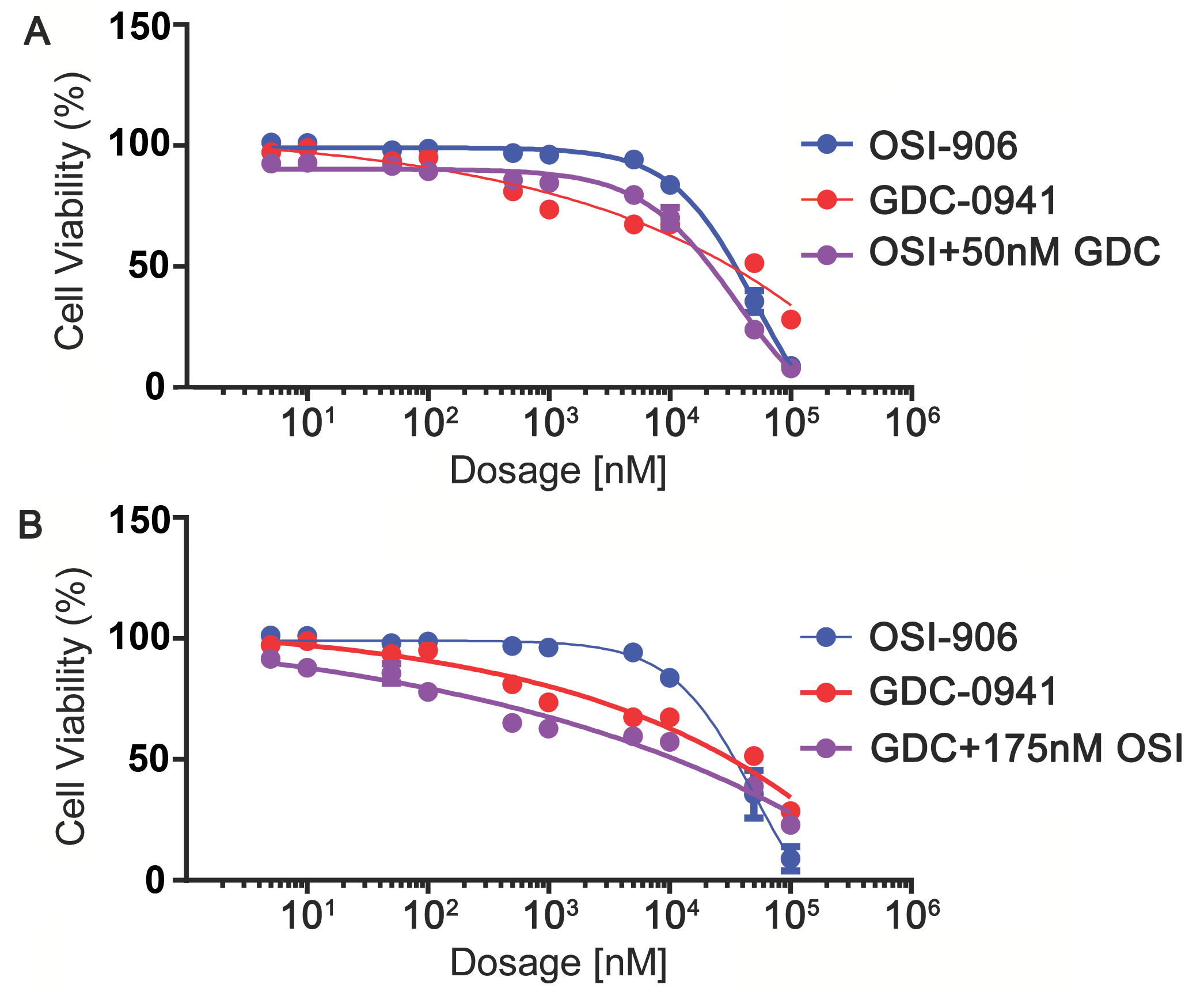

Supplement: Supplementary file 8 — Figure S8. Low dose combination validation results for drug combinations GDC-0941 + OSI-906. Results are based on n = 3 technical replicates with n = 4 replicates per treatment condition. (A) Dose response curve for OSI-906 varied dosage + GDC-0941 low fixed dosage. The response for GDC-0941 at varied dosages is included. (B) Dose response curve for GDC-0941 varied dosage + OSI-906 low fixed dosage. The response for OSI-906 at varied dosages is included. (TIF 10578 kb) [file 12885_2019_5681_MOESM8_ESM.tif]

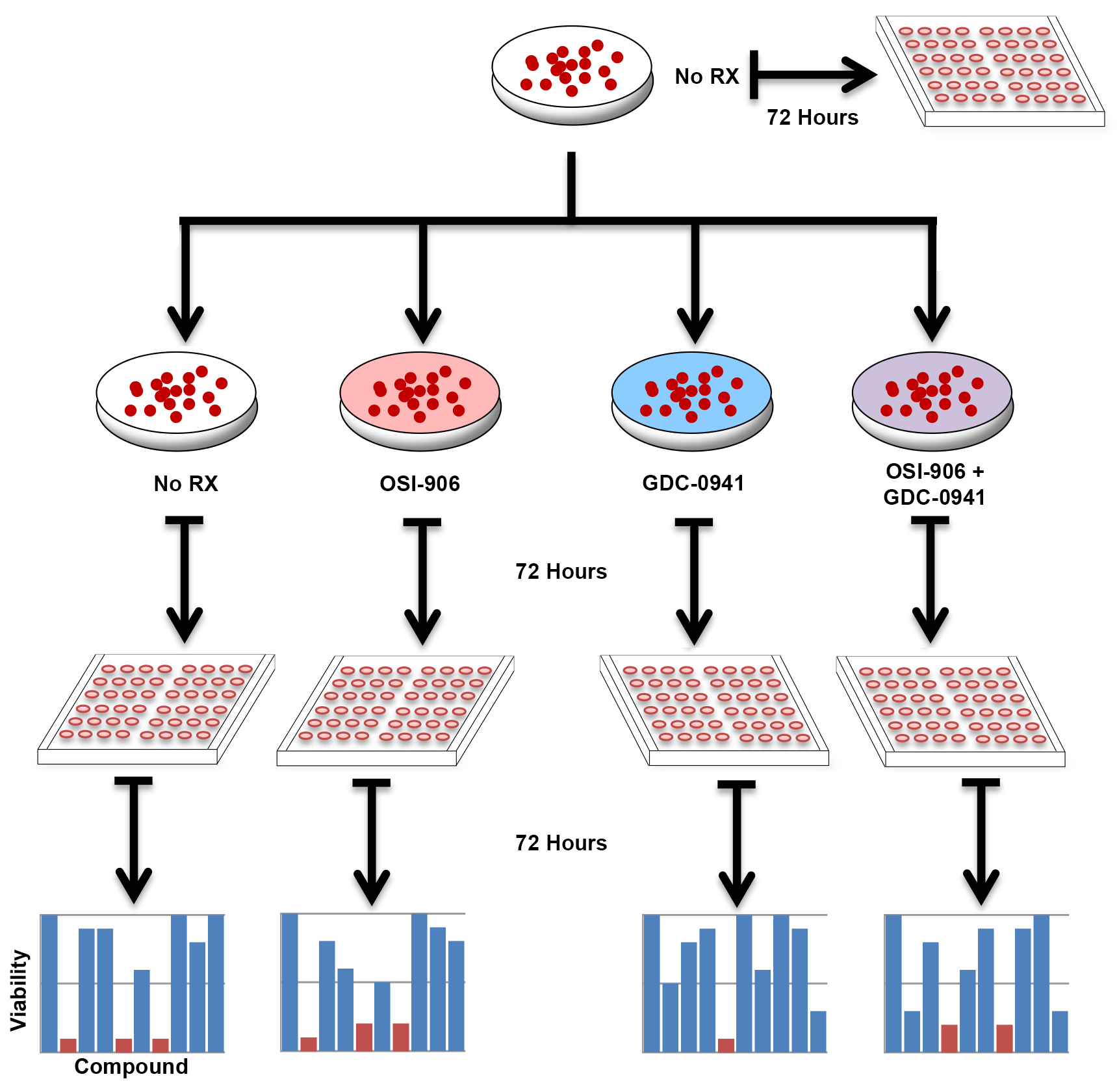

Supplement: Supplementary file 10 — Figure S10. Schematic of PTIM-informed U23674 rewiring experiment. An initial culture of U23674 is screened using the Roche screen. The same culture is used to seed 6 new cultures, which are grown until the cell population is sufficient for drug screening. Five of the 6 cultures were treated using single agents and drug combinations in low dosages (75 nM OSI-906, 50 nM GDC-0941) and one culture was left untreated. After treatment and incubation for 72 h, the compounds were removed the cells were screened using the Roche Orphan Kinome screen. (TIF 8496 kb) [file 12885_2019_5681_MOESM10_ESM.tif]

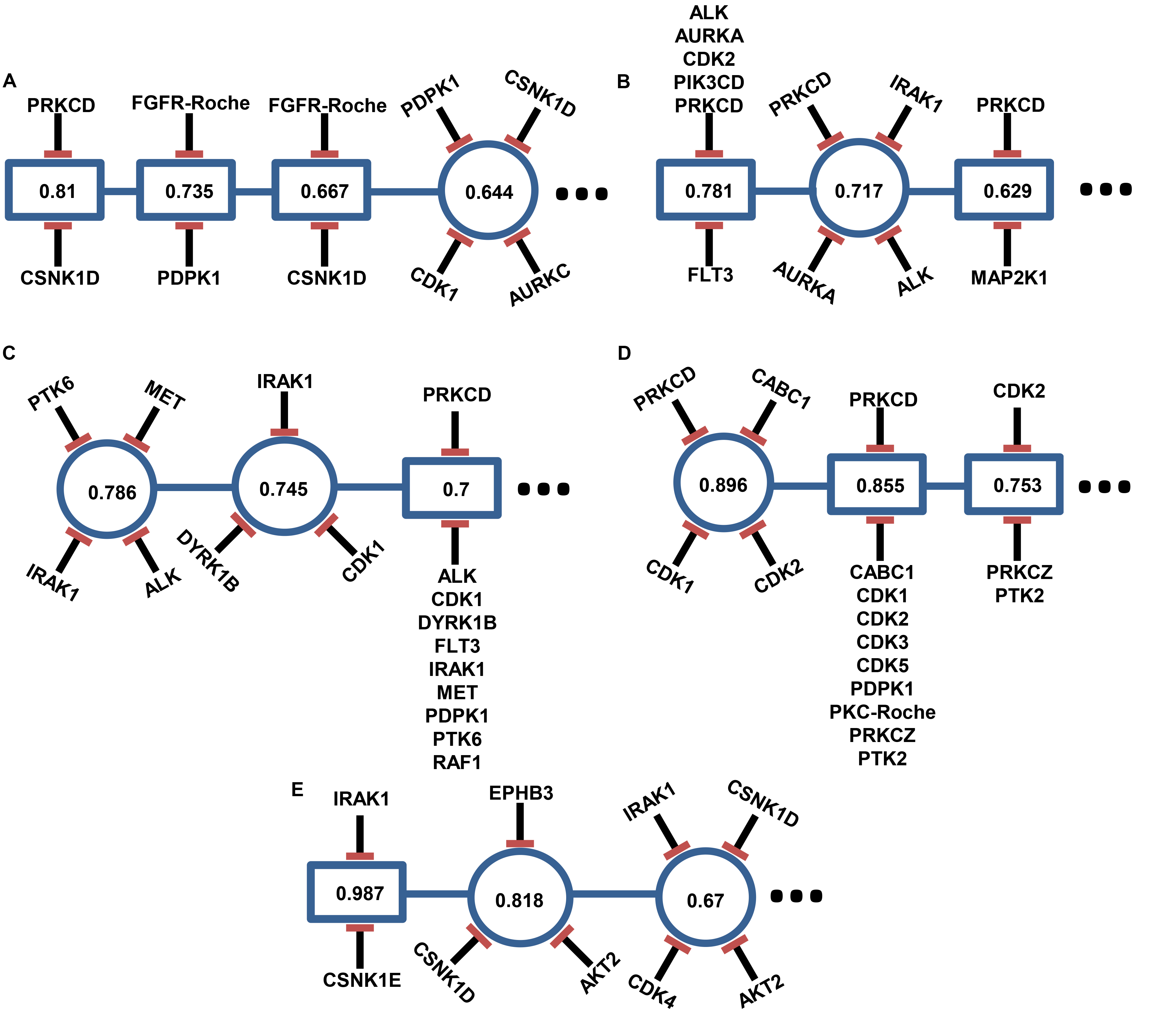

Supplement: Supplementary file 12 — Figure S12. Probabilistic Target Inhibition Map (PTIM) models from U23674 experimental rewiring data. Values in the center of PTIM blocks represent expected scaled sensitivity following inhibition of associated block targets. (A) Untreated initial culture PTIM. (B) Untreated secondaryculture PTIM. (C) OSI-906-treated rewire PTIM. (D) GDC-0941-treated rewire PTIM. (E) OSI-906 + GDC-0941-treated rewire PTIM. (TIF 41768 kb) [file 12885_2019_5681_MOESM12_ESM.tif]

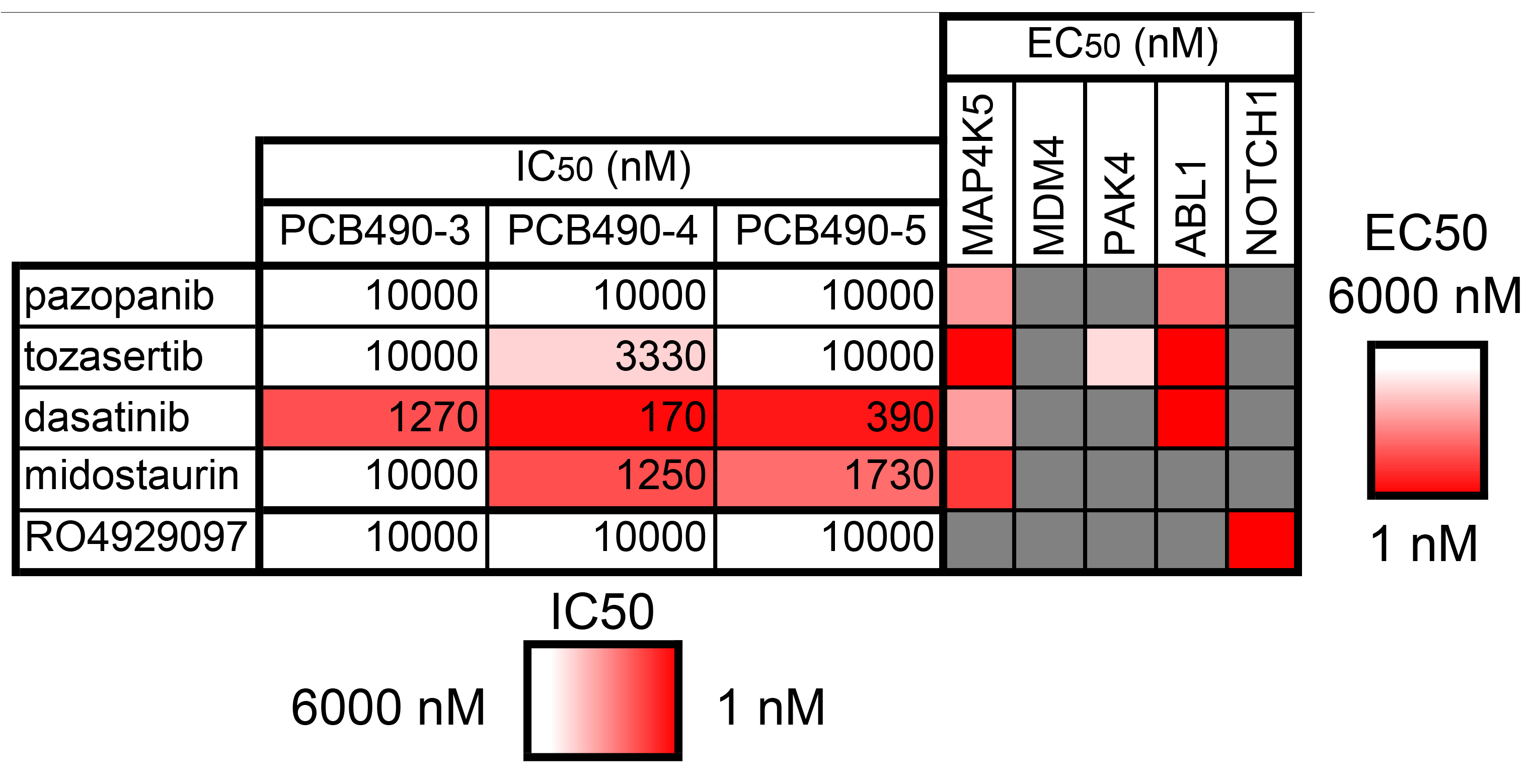

Supplement: Supplementary file 14 — Figure S14. Heat map of IC50 and EC50 values for Pediatric Preclinical Testing Initiative Version 3 drug screen compounds inhibiting mutated and expressed targets in PCB490. Red in the IC50 and EC50 tables indicates low IC50 and EC50 values, respectively. No single target or combination of targets showed uniform efficacy across all PCB490 cultures, suggesting variations alone or in conjunction with transcriptome sequencing would not have identified actionable therapeutic targets. Heat values in the IC50 section of the table represent drug sensitivities as IC50 values, between 1 nM (red) and 6 μM or above (white). Heat values in the EC50 section of the table represent quantified drug-target interaction between chemical agents and gene targets, quantified as 50% inhibitory concentrations between 1 nM (red) and 6 μM or above (white), with grey representing no interaction. (TIF 13895 kb) [file 12885_2019_5681_MOESM14_ESM.tif]
